# Anti-Tumor Potential of Frankincense Essential Oil and Its Nano-Formulation in Breast Cancer: An In Vivo and In Vitro Study

**DOI:** 10.3390/pharmaceutics17040426

**Published:** 2025-03-27

**Authors:** Nouran Mohamed, Hisham Ismail, Ghada M. Nasr, Shaimaa Abdel-Ghany, Borros Arneth, Hussein Sabit

**Affiliations:** 1 Department of Environmental Biotechnology, College of Biotechnology, Misr University for Science and Technology, Giza P.O. Box 77, Egypt; 2 Department of Molecular Diagnostics, Genetic Engineering and Biotechnology Research Institute, University of Sadat City, El Sadat City 32897, Menofia, Egypt; 3 Institute of Laboratory Medicine and Pathobiochemistry, Molecular Diagnostics, Hospital of the Universities of Giessen and Marburg (UKGM), Philipps University Marburg, Baldinger Str., 35043 Marburg, Germany; 4 Institute of Laboratory Medicine and Pathobiochemistry, Molecular Diagnostics, Hospital of the Universities of Giessen and Marburg (UKGM), Justus Liebig University Giessen, 35392 Giessen, Germany; 5 Department of Medical Biotechnology, College of Biotechnology, Misr University for Science and Technology, Giza P.O. Box 77, Egypt

**Keywords:** *Boswellia carterii*, frankincense essential oil, GC/MS, chitosan nanoparticles, breast cancer, female balb/c mice

## Abstract

**Background/Objective:** Breast cancer remains the most common malignancy among women worldwide, contributing to high morbidity and mortality rates. Many anti-cancer drugs have been derived from medicinal plants, and frankincense from *Boswellia carterii* is notable for its anti-inflammatory, anti-neoplastic, and anti-carcinogenic properties. Using gas chromatography/mass spectrometry (GC/MS), 48 components were identified in *B. carterii* essential oil, and the major constituent was α-pinene (35.81%). **Method:** In this study, we investigated the anti-tumor effects of frankincense essential oil (FEO) and its nano-formulation with chitosan (FEO-CSNPs) using in vitro breast cancer models (MCF-7, MDA-MB-231, and 4T1 cells) and in vivo mouse mammary carcinoma (4T1) models (Balb/c). **Results:** The results showed significant reductions in cell viability. At 10 μg/mL, the FEO showed the highest reduction in the C-166 cells, while at 100 μg/mL, the FEO exhibited a stronger cytotoxicity in the MDA-MB-231 and 4T1 cells compared to the FEO-CSNPs and CSNPs. The FEO-CSNPs exhibited cell growth arrest in the S, G2/M, and G1/S phases in the MCF-7, MDA-MB-231, and 4T1 cell lines (36.91%, 23.12%, and 33.58%), in addition to increased apoptosis rates in the MCF-7, MDA-MB-231, and 4T1 cell lines (33.04%, 36.39%, and 42.19%). The wound healing assays revealed a decreased migratory ability in the treated cells. The in vivo experiments in the balb/c mice demonstrated a reduction in the tumor volume, with a histopathological analysis confirming extensive tumor necrosis. Moreover, the FEO and FEO-CSNPs showed notable antioxidant and arginase activity. The gene expression analysis via qPCR indicated the upregulation of tumor suppressor genes and the downregulation of oncogenes. **Conclusions:** These findings suggest that FEO and its nano-formulation, particularly in the form of FEO-CSNPs as an oral formulation, display enhanced efficacy, warranting further preclinical and clinical research to develop innovative treatment strategies.

## 1. Introduction

In 2024, the United States is projected to have 2,001,140 new cancer cases and 611,720 deaths, with these gains threatened by rising incidence rates for 6 of the top 10 cancers; from 2015 to 2019, the incidence increased annually by 0.6–1% for breast, pancreatic, and uterine cancers, and by 2–3% for prostate, liver (in females), kidney, and HPV-associated oral cancers and melanoma [1]. Breast cancer encompasses a diverse spectrum of molecular subtypes and clinical presentations, each carrying unique prognostic implications and varying responses to treatment [2]. In Egypt, breast cancer accounts for 38.8% of malignancies in the female population, with cases estimated to grow from nearly 22,700 in 2020 to almost 46,000 by 2050 [3,4].

Current breast cancer treatments include surgery, chemotherapy, radiation, and hormone therapy. However, these options are increasingly challenged by severe side effects and rising drug resistance [5,6]. This has led to a growing interest in bioactive compounds derived from plant metabolites, which show potential physiological and pharmacological benefits [7].

Frankincense, a resin derived from the gum of Boswellia trees, has a long history in traditional medicine for treating ailments like pain, rheumatoid arthritis, asthma, infections, and cancer [8,9]. Boswellia species, such as *B. sacra* (grown in Oman and Yemen), *B. carterii* and *B. frereana* (grown in Somalia), *B. dalzielii* (Nigeria), and *B. serrata* (grown in India), produce this aromatic resin [10,11].

Extracts from Boswellia species have demonstrated antibacterial [12], antifungal [13], anti-inflammatory [14], and anti-cancer properties [15]. Notably, frankincense oil has shown significant inhibitory effects on various cancer cell types [16]. Preclinical and clinical trials have further indicated frankincense’s anti-cancer potential in breast cancer [17,18].

A common limitation in cancer treatment is the insolubility and limited bioavailability of many drugs and active compounds in tumor environments. Conventional treatments deliver only a fraction of the drug to the tumor, with most dispersing based on the drug’s physicochemical properties to non-target areas [19]. Finding therapies that can actively target tumor sites and enhance the bioavailability of the active compounds is a major goal in cancer treatment [20,21]. Nanocarriers offer a solution, improving the stability, solubility, and bioavailability of therapeutic agents while addressing other drawbacks of conventional medications [22]. The enhanced permeability and retention (EPR) effect in tumors allows nanocarriers to target cancer cells by penetrating the tumor microenvironment. Nanocarriers exploit tumor-associated features, like permeable blood vessels, to improve targeted drug delivery [23]. Nanocarriers can be made from polymers of natural or synthetic origin [24]. One such polymer, chitosan, is commonly used in the food and biomedical industries. This natural polymer, derived from the N-deacetylation of chitin, has notable therapeutic properties, including anti-inflammatory [25], antifungal [26], antibacterial [27], and anti-tumor activities [28].

Chitosan’s properties, including its biodegradability, water solubility, biocompatibility, and non-toxicity, make it an ideal material for drug delivery systems. Its capacity for adhesion to mucosal surfaces [29,30] and the electrostatic interaction between its positively charged structure and negatively charged cell membranes further enhance its suitability in therapeutic applications [31]. These properties have been leveraged to engineer FEO-CSNPs, which fall within the optimal size range for exploiting the enhanced permeability and retention (EPR) effect, making them well suited for passive tumor targeting. Additionally, the stability and surface properties of FEO-CSNPs, derived from chitosan’s unique characteristics, further enhance their potential as an effective passive targeting system.

In this study, we investigated the anti-cancer potential of *B. carterii* essential oil, either in its free or chitosan-loaded form, against breast cancer both in vitro and in vivo.

## 2. Materials and Methods

### 2.1. Plant Material

Frankincense pure essential oil of *Boswellia carterii* was purchased from Harraz Co., Cairo, Egypt. The purity of the oil was 99.6%.

### 2.2. Gas Chromatography/Mass Spectroscopy (GC-MS) Analysis

Commercial *B. carterii* essential oil was analyzed using Shimadzu GCMS-QP2010 (Koyoto, Japan) equipped with Rtx-5MS fused silica bonded column (30 m × 0.25 mm i.d. × 0.25 µm film thickness; Restek, USA) and a split–splitless injector. The initial column temperature was kept at 45 °C for 2 min (isothermal) and then programmed to increase to 300 °C at a rate of 5 °C/min, where it was held constant for 5 min (isothermal). The injector temperature was set to 250 °C, and the helium carrier gas flow rate was 1.41 mL/min. Mass spectra were recorded under the following conditions: filament emission current, 60 mA; ionization voltage, 70 eV; and ion source temperature, 200 °C. Diluted samples (1% *v*/*v*) were injected in split mode with a split ratio of 1:15.

### 2.3. FEO-CSNP Formulation

Chitosan (CS) (high molecular weight: 310,000–375,000 Da) was purchased from El-Sharq Co., Cairo, Egypt. After minor adjustments, ionic gelation of CS with tripolyphosphate (TPP) anions was used to create chitosan nanoparticles (CSNPs), following the method first described by [32]. Chitosan solution was prepared by dissolving purified chitosan with sonication in 1% (*w*/*v*) acetic acid solution until the solution was transparent. Once dissolved, the chitosan solution was diluted with deionized water to provide chitosan solution with a concentration (*w*/*v*) of 0.10%. Deionized water was used to dissolve tripolyphosphate with a concentration (*w*/*v*) of 1%. Under strong magnetic stirring at room temperature, a concentration of 1% aqueous solution of TPP was added dropwise to the CS solution. CSNPs were formed spontaneously. To separate the nanoparticles from larger particles or aggregates, the nanoparticle suspension was centrifuged at 10,000× *g* for 30 min at room temperature. Alternatively, the suspension was freeze-dried to obtain powder samples. Sediment and supernatant were separated following centrifugation by carefully removing the supernatant layer. FEO-CSNPs were formulated by loading 200 µL FEO in 5 mL ethanol on the nanoparticles pellet for 24 h. Finally, the FEO-CSNPs were centrifuged at 10,000 rpm at 4 °C for 10 min, and the resulting residue was separated and dried at room temperature.

### 2.4. Characterization of the Nanomaterial

#### 2.4.1. Transmission Electron Microscopy (TEM)

The morphological characteristics of nanoparticles were examined using a high-resolution transmission electron microscope (Model: JEM 2100 LB_6,_ Akishima, Tokyo, Japan) after spreading one drop of the nanoparticle solution over a copper grid coated with carbon and stained with 2% (*w*/*v*) phosphotungstic acid. The samples were subjected to TEM examination using an accelerating voltage of 200 kV after drying at room temperature.

#### 2.4.2. Dynamic Light Scattering (DLS) and Zeta Potential

The average particle size of nanoparticles was measured as described by [33]. Particle size distribution and zeta potential of CSNPs were measured through DLS with Zetasizer Nano S (Malvern, UK). Nanoparticles dispersed in deionized distilled water (2 mg of sample was dissolved in 5 mL of deionized water, followed by sonication in a sonics Vibra cell sonicator) were used in the analysis, which was conducted at 25 °C and with a scattering angle of 90°. A polydispersity index is used to describe the nanoparticles’ particle size distribution (PDI).

#### 2.4.3. Fourier Transform Infrared (FT-IR) Spectroscopy

An infrared spectrometer (FT-IR) (JASCO FT/IR-4100, JASCO, Tokyo, Japan) was used to determine the FT-IR spectra of free CSNPs, free frankincense essential oil, and FEO-CSNPs. With 16 scans at a resolution of 0.4 cm^−1^, the spectra were acquired in the range between 500 and 4000 cm^−1^.

#### 2.4.4. Encapsulation Efficiency and Drug-Loading Capacity

Colorimetric analysis was used to calculate the FEO loading capacity (LC%) and entrapment efficiency (EE%). Briefly, the freeze-dried FEO-CSNPs were vigorously stirred in 5% (*v*/*v*) HCl for 10 min. Following the removal of the residual pellets, the absorbance at λ = 235 nm (UV-VIS spectrophotometer, Shimadzu UV1800) was used to calculate the FEO concentration in the supernatant. Then, EE% and LC% were calculated using the following equations:

$$EE\%=\frac{Amount\;of\;loaded\;FEO}{The\;initial\;amount\;of\;FEO}\times 100$$

$$LC\%=\frac{Amount\;of\;loaded\;FEO}{Weight\;of\;dried\;CSNPs}\times 100$$

#### 2.4.5. In Vitro Drug Release Study

The in vitro release rate of FEO from FEO-CSNPs was evaluated utilizing the dialysis bag method. Briefly, 2 mL of the suspension was added to a dialysis bag (12–14 KD cut off, Sigma Aldrich, Darmstadt, Germany). The dialysis bag was sealed properly both from top and bottom and inserted into 20 mL of phosphate-buffer saline (PBS) with pH 7.4, pH 6.8, and pH 1.2 individually for 24 h in properly closed flasks. The whole system was fixed in a shaking incubator (Jeio tech SI-300, Seoul, Republic of Korea), rotating at 100 rpm with a temperature adjusted to 37 °C. At predetermined time intervals, 2 mL samples were withdrawn from the release medium and immediately replaced with another 2 mL of warmed fresh buffer for 24 h. The withdrawn samples were measured using a UV-Spectrophotometer (JENWAY 6305, JENWAY, Staffordshire, UK) at wavelength 235 nm.

### 2.5. Cell Line Maintenance and Drug Treatment

The murine triple-negative breast cancer cell line (4T1), human breast adenocarcinoma (MCF-7), human triple-negative breast cancer (MDA-MB-231), and the normal mouse endothelial cell line (C-166) were sourced from NAWAH Scientific, Cairo, Egypt. These cell lines were cultured in Dulbecco’s Modified Eagle’s Medium (DMEM), supplemented with 10% fetal bovine serum (FBS; Sigma-Aldrich) and 2% penicillin–streptomycin (Life Technologies, Grand Island, NY, USA). The cells were maintained under controlled conditions at 37 °C in a humidified atmosphere containing 95% air and 5% CO_2_. To prepare for experiments, the cells were dissociated using trypsin and seeded at a density of 1 × 10^4^ cells/mL into 9-well polystyrene culture plates. They were subsequently exposed to various treatments, including frankincense essential oil (FEO), FEO loaded on chitosan nanoparticles (FEO-CSNPs), and chitosan nanoparticles (CSNPs), at a concentration of 10 μL/3 mL. After 24 h of treatment, cell viability was assessed using the sulforhodamine B (SRB) assay, a standard method for determining cytotoxicity and cellular proliferation.

### 2.6. Cytotoxicity Assay

Cell viability was evaluated using the SRB assay. A 100 μL aliquot of cell suspension (5 × 10^3^ cells per well) was seeded onto 96-well plates and incubated in complete growth media for 24 h to allow cell adherence. Subsequently, cells were treated with 100 μL of media containing varying concentrations of the test compounds (10 μg and 100 μg) for the designated exposure time. Following drug treatment, cells were fixed by replacing the media with 150 μL of cold 10% trichloroacetic acid (TCA), and the plates were incubated at 4 °C for 1 h. After removing the TCA solution, the wells were thoroughly washed five times with distilled water. Next, 70 μL of SRB solution (0.4% *w*/*v*) was added to each well, and the plates were incubated in the dark at room temperature for 10 min. The excess dye was removed by washing the plates three times with 1% acetic acid, followed by air drying overnight. To solubilize the SRB-stained proteins, 150 μL of 10 mM TRIS buffer was added to each well, and the absorbance was measured at 540 nm using the Infinite F50 microplate reader (TECAN, Männedorf, Switzerland). This method enables precise quantification of cell viability based on protein content.

### 2.7. Cell Cycle Distribution

Cells (4 × 10^4^) were plated into 12-well plates and subjected to the specified treatments. After 24 h, the cells were harvested using cold 70% ethanol in PBS for five min at 600 rpm. Following this, the cells were centrifuged again at 600 rpm for five min and stored at 4 °C for two hours. The samples were then treated with 50 μg/mL propidium iodide (PI), 0.1% Triton X-100, and 50 μg/mL RNAse for 25 min. This was followed by incubation at room temperature, in the dark, to protect the PI from photobleaching. The fluorescence of PI was subsequently measured using a FACScan flow cytometer (BD FACSCalibur^TM^, BD Biosciences, Milpitas, CA, USA), and the resulting data were analyzed to determine cell cycle distribution in both treated and untreated cells. This methodology allowed for precise monitoring of cell cycle progression and the impact of the applied treatments.

### 2.8. Apoptosis Detection

Cells were seeded into 12-well tissue culture plates and treated for 24 h with the appropriate experimental conditions. Apoptotic cells were identified using Annexin V-fluorescein isothiocyanate (FITC) and propidium iodide (PI) staining. Briefly, after collecting both treated and control cells, they were resuspended in 100 μL of Annexin V binding buffer mixed with 5 μL of Annexin V Alexa Fluor 488. The samples were incubated in the dark for 15 min to prevent photobleaching. Following incubation, 4 μL of PI, diluted in a 1:10 ratio in 1× Annexin V binding buffer, was added and incubated for an additional 15 min under the same conditions. The cells were then washed with 500 μL of Annexin V binding buffer before analysis. Flow cytometry (BD FACSCalibur^TM^) was employed to visualize the stained cells. Annexin V-FITC binding was detected at an excitation wavelength of 488 nm and an emission wavelength of 530 nm using the FITC signal detector (FL1). At the same time, PI staining was assessed using the phycoerythrin emission signal detector (FL2). This procedure ensured accurate quantification of apoptotic cell populations.

### 2.9. Wound Healing Assay

The wound healing assay was conducted to assess the migratory capacity of tumor cells. Specifically, 1 × 10^4^ cells were seeded into a 9-well plate and treated with various conditions, allowing them to grow until reaching 70–80% confluence. A wound was generated by carefully scratching the cell monolayer with a sterile 200 μL pipette tip. Post-wound creation, cell migration into the gap was monitored at multiple time points. The extent of migration was documented through photomicrographs captured using an inverted microscope at ×100 magnification, enabling precise observation of cell movement toward the wounded area. This assay provided crucial insights into the impact of different treatments on the cells’ migratory behavior, a key indicator of metastatic potential.

### 2.10. In Vivo Studies

Fifty-five female Balb/c mice, each weighing approximately 25 g, were sourced from the Animal House at the Faculty of Veterinary Medicine, Cairo University, Giza, Egypt. The mice were housed under standard conditions at the University Research Animal Facility (URAF), following the guidelines for ethical animal care. The Institutional Animal Care and Use Committee (IACUC) of Cairo University approved the study’s protocol, ensuring strict adherence to ethical practices. Mice were maintained in a pathogen-free environment with free access to food and water, a controlled temperature of 22 ± 0.5 °C, and a 12 h light/dark cycle.

After a one-week acclimatization period, the mice were randomly assigned into five groups (n = 11). Group I served as the negative control, receiving only 100 μL of PBS, while Groups II to V were injected with 1 × 10^5^ 4T1 cells suspended in 100 μL culture media and inoculated into the fourth pair of mammary glands. Tumor growth was closely monitored over 10 days with bidirectional measurements using electronic calipers. Group II, the positive control, comprised tumor-bearing mice. Group III received 6 μL/g of Frankincense essential oil (FEO), Group IV received 12 μL/g of FEO loaded on chitosan nanoparticles (FEO-CSNPs), and Group V received 12 μL/g of chitosan nanoparticles (CSNPs) daily via oral gavage for 21 days.

Morbidity and mortality were tracked daily. Tumor volume (Tv) was calculated using the formula Tv = L × W × W/2, where L represents the shorter measurement, and W is the longer measurement. After 21 days of treatment, mice were anesthetized with ketamine (80 mg) and xylazine (8 mg) for blood collection. All animals were humanely sacrificed via cervical dislocation. Mammary tumors were harvested, with portions preserved in 10% formalin for histopathological evaluation and QIAzol^®^ lysis reagent for molecular studies and stored at −20 °C for biochemical analyses.

#### 2.10.1. Histopathological Analysis

Histopathological sections from both treated and untreated mice mammary glands were meticulously prepared using an ultra-microtome. Standard histological techniques were employed, beginning with tissue fixation in formalin to preserve cellular structures, followed by paraffin embedding. Tissue blocks were sectioned into thin slices, approximately 3–5 μm thick, using the microtome. These sections were subsequently stained with hematoxylin and eosin (H&E) to visualize cellular morphology and tissue architecture. The stained slides were examined under a light microscope to assess any histological alterations, enabling the comparison of pathological changes between the treatment and control groups.

#### 2.10.2. Arginase Activity

Tissue homogenates (50 µL) or 1 × 10^6^ cells were incubated with 0.1% Triton X-100 and then mixed with Tris-HCl and MnCl₂ for activation at 56 °C for 10 min. After incubating with L-arginine (pH 9.7) at 37 °C for 60–120 min, the reaction was stopped using a sulfuric acid–phosphoric acid solution. The final mixture was combined with isonitrosopropiophenone and heated at 95 °C for 30 min. Urea concentration was determined via spectrophotometry at 540 nm, with one unit of enzyme activity defined as the amount catalyzing 1 μmol of urea per minute.

#### 2.10.3. GST Activity

Glutathione-S-transferase (GST) activity was assessed at 25 °C using 100 mM sodium phosphate buffer (pH 6.5) with 1 mM EDTA. The substrates, 1 mM CDNB and 1 mM reduced glutathione, were used to initiate the reaction, and the absorbance change was measured at 340 nm. Enzyme activity was calculated based on the molar absorption coefficient of 9.6 mM^−1^ cm^−1^, defining one unit of GST activity as the amount of enzyme that catalyzes the formation of 1 μmol of product per minute under these conditions.

#### 2.10.4. Glutathione (GSH) Activity

Tissues were homogenized in 4 volumes of ice-cold 0.1 M potassium phosphate buffer (pH 7.4) with 1 mM EDTA. The homogenate was vortexed and mixed with an equal volume of 2.5% sulfosalicylic acid, then cooled for 5 min. After centrifugation at 4 °C for 10 min at 3000× *g*, the supernatant was collected. The reaction was initiated by adding 0.5 mL supernatant, 2 mL potassium phosphate buffer (pH 7.2), and 0.5 mL DTNB (0.04%) to the cuvette. The formation of 5-thio-nitrobenzoic acid was measured at 412 nm, and glutathione content was calculated from a standard curve expressed as moles/g wet tissue.

#### 2.10.5. MDA Activity

The tissues underwent perfusion and homogenization in 50 mM potassium phosphate buffer (pH 7) before being centrifuged for 5 min at 4 °C at 14,000 rpm. To 100 μL of the supernatant, 1400 μL of 15% trichloro acetic acid containing 0.375% thiobarbituric acid and 14 μL of butylated hydroxytoluene (20 mg/mL absolute alcohol) were added to form the reaction mixture. Samples were centrifuged at 14,000 rpm for 5 min at 4 °C after being heated at 100 °C for 15 min in a water bath. The absorbance of the supernatants was measured at 532 nm using a spectrophotometer to quantify MDA concentrations.

### 2.11. Gene Expression Analysis

Total RNA was extracted from both treated and untreated cells using the GeneJET RNA Purification Kit (Thermo Fisher Scientific, Waltham, MA, USA, Cat. No. K0731). Genomic DNA was eliminated using DNase I, RNase-free kit (Thermo Fisher Scientific, USA, Cat. No. EN0521). RNA concentration and purity were assessed using a NanoDrop spectrophotometer (IMPLEN, Munich, Germany). Complementary DNA (cDNA) was synthesized from 1 μg of RNA by combining 1 μL RT Primer Mix and 1 μL Quantiscript Reverse Transcriptase using the QuantiTech Reverse Transcription Kit (QIAGEN, Cat. No. 205311). The reaction was incubated at 42 °C for 15 min, followed by 95 °C for 3 min. Quantitative real-time PCR (qPCR) was performed to examine the expression of PIK3CA, CCND1, HER2, STAT3, WNT1, KRAS, TP53, CDH1, and PTEN genes (Table 1), using GAPDH as the reference gene. Primers were designed using Primer-BLAST (NCBI). Each 20 μL qPCR reaction contained 10 μL of HERAPLUS SYBR^®^ Green Master Mix, 1 μL of each primer (forward and reverse), 2 μL cDNA, and 6 μL DNase-free water. Amplification was performed on a StepOne Plus thermal cycler (ABS, London, UK) with the following conditions: 95 °C for 5 min, followed by 40 cycles of 95 °C for 1 min, 57 °C for 25 s, and 72 °C for 1 min. Melting curves were analyzed to confirm specific amplification. Gene expression changes were quantified using the 2^−ΔΔCt^ method, with GAPDH serving as the internal control. Relative fold changes were calculated based on the ΔΔCt values between treated and control groups.

### 2.12. Statistical Analysis

The experimental data are expressed as mean ± standard error (SE), with results based on three independent replicates to ensure reproducibility. Statistical comparisons between treated and untreated groups were performed using one-way analysis of variance (ANOVA), followed by post hoc tests, where appropriate. A threshold of *p* < 0.05 was considered statistically significant. For gene expression analyses, Student’s *t*-test was employed to assess differences between treatment and control groups, ensuring a robust evaluation of the experimental outcomes. This statistical approach allows for accurate interpretation of the data and validation of significant findings in gene expression and treatment effects.

## 3. Results

### 3.1. GC-MS Analysis

**Figure 1:**
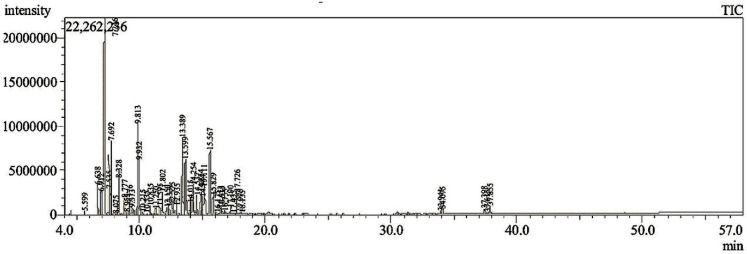
GC-MS chromatogram of the commercial frankincense essential oil derived from *Boswellia carterii*.

The GC/MS chromatogram of the commercial frankincense essential oil revealed the presence of 48 compounds, as shown in (Figure 1) and (Table 2), representing monoterpenes along with oxygenated terpenoids, which were identified by comparing the fragmentation patterns in the resulting mass spectra (provided in the Supplementary File) with those reported in the literature and by using the NIST mass spectral database of the gas chromatograph’s computer. Among the identified compounds, α-Pinene (35.81%), Sabinol (6.47%), trans-Verbenol (5.55%), m-Cymene (5.32%), Verbenone (4.74%), Limonene (2.96%), 2,4(10)-Thujadiene (2.95%), α-Phellandren-8-ol (2.86%), α-thujene (2.85%), Myrtenal (2.74%), β-pinene (2.36%), and Camphene (2.31%) were the ones with the highest relative concentrations (area %).

### 3.2. Characterization of Nanomaterial

#### 3.2.1. Transmission Electron Microscopy (TEM)

**Figure 2:**
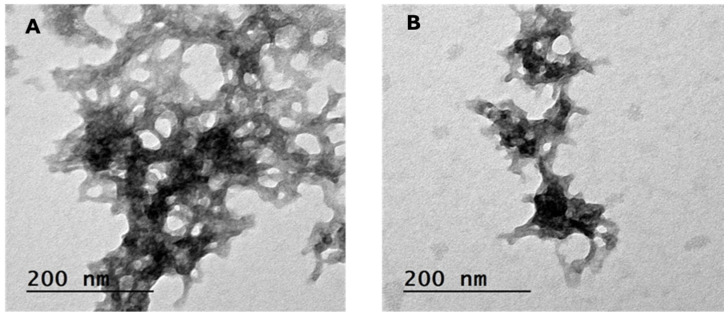
Transmission electron microscopy (TEM) images of (**A**) Chitosan nanoparticles (CSNPs) and (**B**) frankincense essential oil-loaded chitosan nanoparticles (FEO-CSNPs). CSNPs exhibit a regular spherical morphology with an average size of 16.05 nm, while FEO-CSNPs appear larger with a more clustered structure, having an average size of 26.26 nm.

The TEM analysis reveals distinct structural differences between the CSNPs and FEO-CSNPs. The chitosan nanoparticles are uniformly spherical with smooth surfaces, reflecting the stability and regularity of the nanocarrier (Figure 2A). In contrast, the FEO-loaded CSNPs are larger and exhibit a more irregular, clustered appearance (Figure 2B). This increase in size and change in morphology may be attributed to the encapsulation of frankincense essential oil, potentially leading to nanoparticle aggregation or changes in surface properties.

#### 3.2.2. Dynamic Light Scattering and Zeta Potential

**Figure 3:**
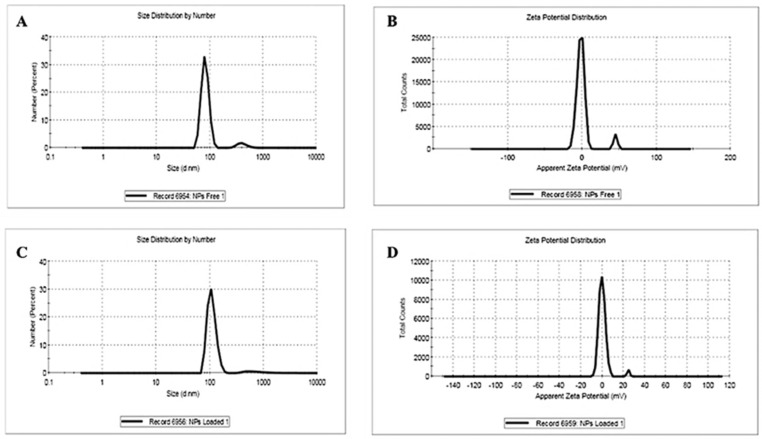
Size distribution and zeta potential analysis of chitosan nanoparticles (CSNPs) and frankincense essential oil-loaded chitosan nanoparticles (FEO-CSNPs). (**A**) Size distribution of CSNPs with a main peak at 82.28 d.nm. (**B**) Zeta potential distribution of CSNPs. (**C**) Size distribution of FEO-CSNPs with a main peak at 109.3 d.nm. (**D**) Zeta potential distribution of FEO-CSNPs.

The dynamic light scattering (DLS) analysis shows that CSNPs have a hydrodynamic size range between 69.72 and 151.08 d.nm, with a predominant peak at 82.28 d.nm and a polydispersity index (PDI) of 0.54, indicating moderate size uniformity (Figure 3). Their zeta potential was recorded at 1.06 mV, suggesting relatively low surface charge. Upon loading frankincense essential oil, the FEO-CSNPs displayed an increased hydrodynamic size range (82.19 to 312.06 d.nm) with a larger main peak at 109.3 d.nm and a PDI of 0.62, indicating a slightly broader size distribution. The zeta potential of the FEO-CSNPs decreased to 0.59 mV, which might reflect changes in surface charge due to the incorporation of the oil, potentially affecting nanoparticle stability and interaction.

#### 3.2.3. Fourier Transform Infrared Spectroscopic (FTIR)

**Figure 4:**
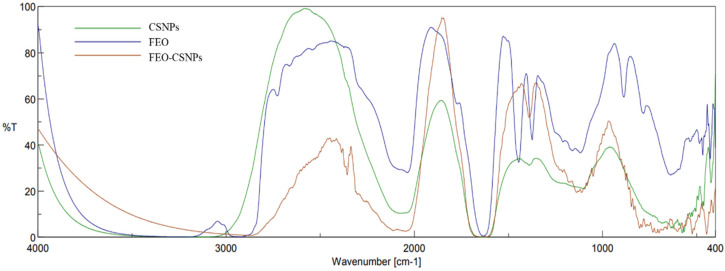
FTIR spectra comparing chitosan nanoparticles (CSNPs), frankincense essential oil (FEO), and FEO-loaded CSNPs. The spectra show characteristic functional group peaks, demonstrating the interaction between CSNPs and FEO after encapsulation, as observed by changes in peak intensities and shifts in wavenumbers.

The FTIR spectra reveal distinct absorption bands corresponding to the CSNPs, FEO, and FEO-loaded CSNPs (Figure 4). The characteristic peaks of CSNPs, such as the strong absorption around 3400 cm^−1^ (O-H and N-H stretching), remain prominent, but with a shift in the FEO-CSNP spectrum, indicating interactions between the chitosan matrix and FEO. Additionally, peaks associated with C-H stretching (~2900 cm^−1^) and carbonyl groups (~1700 cm^−1^) in FEO show changes in intensity and position after encapsulation, further supporting the successful integration of FEO into the nanoparticle structure. These spectral shifts and changes in intensity suggest the formation of a stable FEO-CSNP complex, potentially enhancing the bioactivity of the essential oil when encapsulated. 

#### 3.2.4. EE, LC, and In Vitro Drug Release

**Figure 5:**
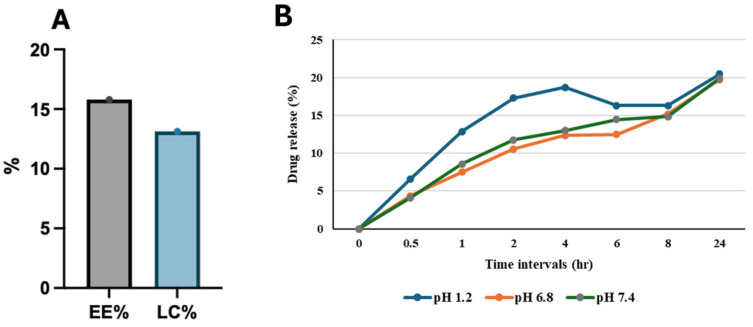
EE, LC, and in vitro release profile. (**A**) EE and LC of the drug. (**B**) In vitro release profile of frankincense essential oil-loaded chitosan nanoparticles (FEO-CSNPs) under different pH conditions (1.2, 6.8, and 7.4). The drug releases most readily in acidic conditions (pH 1.2) initially, stabilizes at 4–8 h, and shows steady release at pH 6.8 and 7.4. By 24 h, all pH levels converge (~20%), indicating a controlled-release formulation for consistent delivery across different pH environments.

According to the previous two equations, the EE% and LC% were 15.794% and 13.125%, respectively (Figure 5A). The release profile of FEO-CSNPs shows a controlled-release design that ensures consistent delivery across various pH conditions (Figure 5B). At 0.5–1 h, drug release at pH 1.2 is higher compared to that at pH 6.8 and pH 7.4. This suggests that the drug dissolves more rapidly in acidic conditions during the initial phase.

At 4–8 h, the acidic medium (pH 1.2) shows a plateau or slight reduction in drug release, which might indicate saturation or slower dissolution at this stage, while pH 6.8 and 7.4 conditions show continuous increases, suggesting that the drug is more stable and released consistently in neutral to slightly acidic environments.

At 24 h, all three pH conditions converge, showing nearly identical drug release (~20%). This indicates that the formulation is designed to release the drug completely, irrespective of pH, over 24 h.

### 3.3. Cytotoxicity Assay

**Figure 6:**
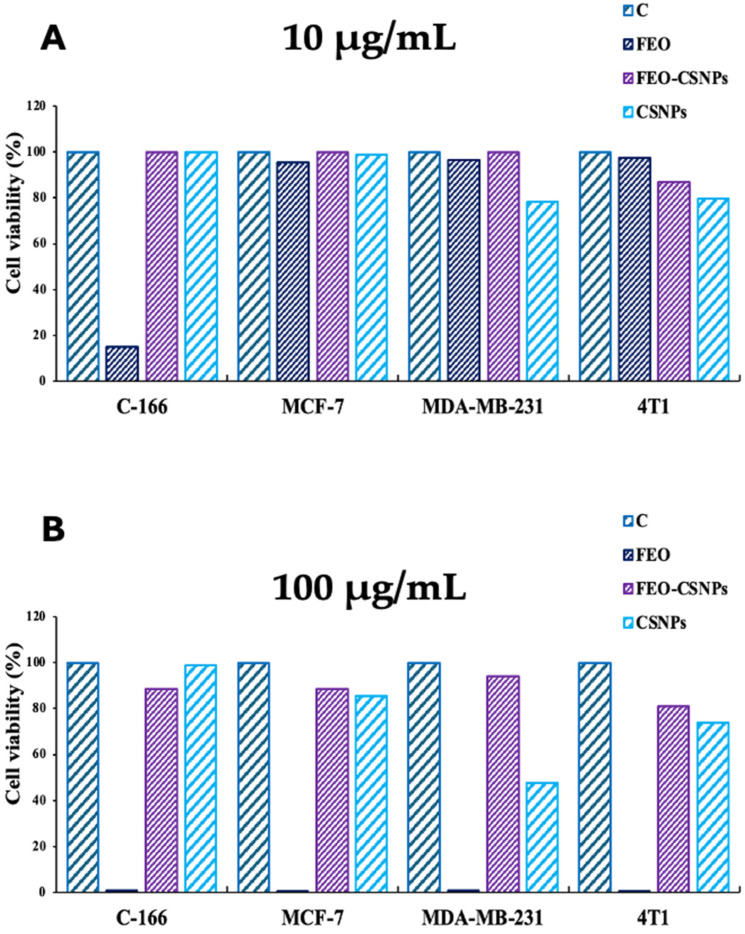
Comparison of cell viability percentages of C-166, MCF-7, MDA-MB-231, and 4T1 cells treated with frankincense essential oil (FEO), FEO-loaded chitosan nanoparticles (FEO-CSNPs), and chitosan nanoparticles (CSNPs) at concentrations of 10 μg/mL (**A**) and 100 μg/mL (**B**). The figure highlights a dose-dependent reduction in cell viability, particularly at higher concentrations of FEO and its nano-formulation in cancer cell lines.

The cytotoxicity results demonstrate that the treatments induced a reduction in cell viability across different cell lines. At 10 μg/mL, minimal differences in cell viability were observed among the treatments, particularly with FEO alone showing the highest reduction in the C-166 cell line (Figure 6A). However, at the higher concentration of 100 μg/mL, the treatments exhibited more pronounced cytotoxic effects, especially in the MDA-MB-231 and 4T1 cells, where FEO significantly lowered the cell viability, outperforming both the FEO-CSNP and CSNP treatments (Figure 6B).

### 3.4. Cell Cycle Distribution

**Figure 7:**
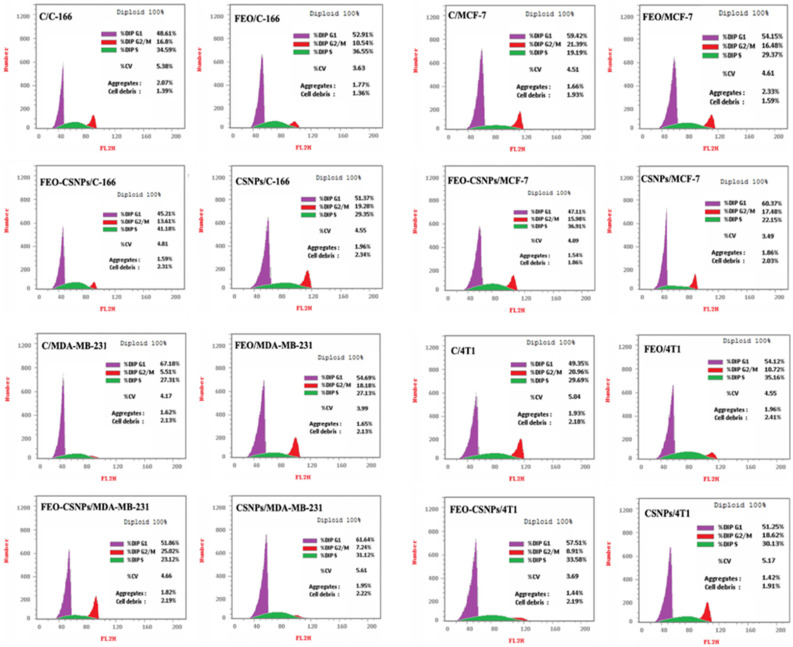
Flow cytometry analysis illustrating the impact of frankincense essential oil (FEO), FEO-loaded chitosan nanoparticles (FEO-CSNPs), and chitosan nanoparticles (CSNPs) on the cell cycle phases in C-166, MCF-7, MDA-MB-231, and 4T1 cell lines after 24 h of treatment. In C-166 cells, treatments caused an accumulation in the S phase, indicating inhibition of DNA replication (36.55%, 41.18%, and 29.35% for FEO, FEO-CSNPs, and CSNPs, respectively). Similar S-phase arrest was seen in MCF-7 cells (29.37%, 36.91%, and 22.15%). In MDA-MB-231 cells, G2/M phase arrest was prominent (18.18%, 25.02%, and 7.24%), suggesting disruption of mitosis. In contrast, 4T1 cells predominantly exhibited G1/S phase arrest (54.12%, 57.51%, and 51.25%), indicating interference with DNA synthesis initiation. These results reflect the differential impact of the treatments on various cell cycle phases depending on the cell line.

The flow cytometry results provide compelling evidence of the distinct effects of FEO, FEO-CSNPs, and CSNPs on cell cycle progression across various cell lines (Figure 7). The C-166 and MCF-7 cells exhibited significant arrest in the S phase, reflecting inhibited DNA synthesis and cell cycle progression. This disruption in the S phase could limit cell proliferation by preventing the cells from entering the G2 phase, effectively halting mitosis. On the other hand, the arrest of the MDA-MB-231 cells in the G2/M phase suggests that these treatments impede cell division, a critical step for mitotic completion, potentially leading to cell death or senescence. In the 4T1 cell line, the G1/S arrest suggests early inhibition in the cell cycle, possibly due to interference in DNA replication initiation. These findings collectively indicate that the treatments effectively target distinct cell cycle checkpoints in various cell lines, pointing to their potential as targeted therapies in cancer treatment.

### 3.5. Apoptosis Detection

**Figure 8:**
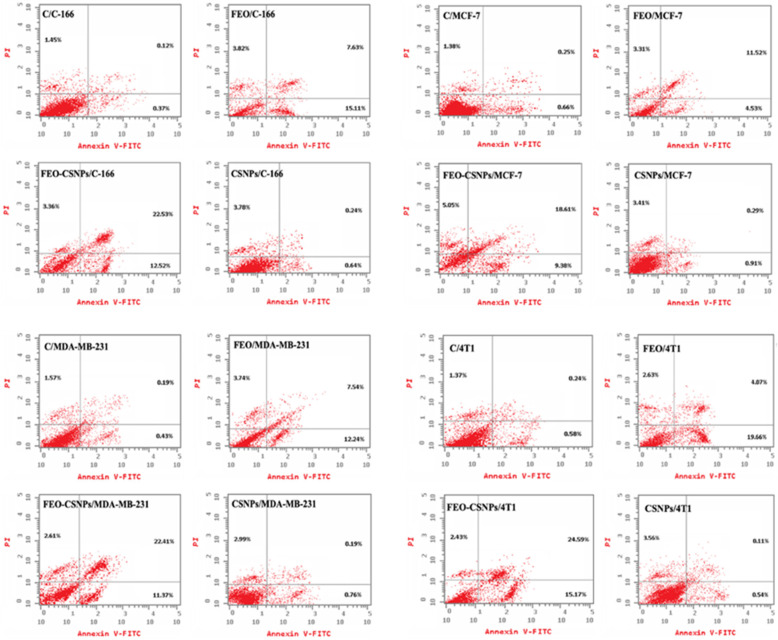
The apoptosis levels of C-166, MCF-7, MDA-MB-231, and 4T1 cells were assessed using Annexin-V-FITC/PI staining after treatment with FEO, FEO-loaded chitosan nanoparticles (FEO-CSNPs), and chitosan nanoparticles (CSNPs) for 24 h. The FEO-CSNP treatment exhibited the highest impact on apoptosis across all cell lines, with notable percentages of total apoptosis observed, as compared to untreated controls: 38.41% in C-166, 33.04% in MCF-7, 36.39% in MDA-MB-231, and 42.19% in 4T1 cells. These findings demonstrate the potential of FEO-CSNPs in inducing programmed cell death in different cancer cell lines, contributing to their anti-cancer properties.

The apoptosis detection analysis through Annexin-V-FITC/PI staining revealed significant cell death induced by FEO-CSNPs in the cancer cell lines tested (Figure 8). Notably, the combination of FEO with chitosan nanoparticles resulted in an enhanced apoptotic effect compared to controls and single treatments, as seen in the high percentages of apoptotic cells, particularly in the 4T1 cells, which showed over 42% total apoptosis. This suggests a synergistic potential of FEO-CSNPs in promoting cell death mechanisms, making it a promising therapeutic option for breast cancer treatment. The increased apoptosis of the MCF-7, MDA-MB-231, and 4T1 cells further reinforces the efficacy of the nano-formulation in targeting various breast cancer subtypes.

### 3.6. Wound Healing Assay

**Figure 9:**
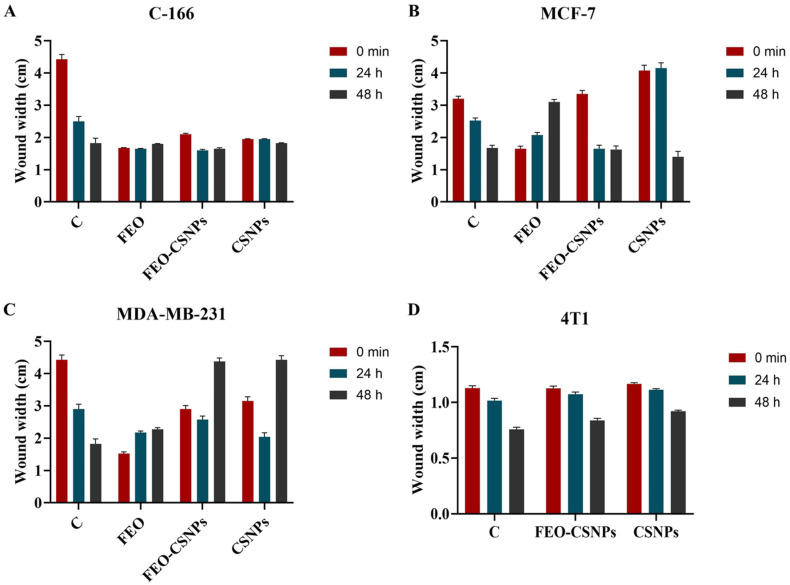
Wound healing responses of breast cancer cell lines following exposure to the treatments. (**A**) Effect of the treatments on wound width of C-166 cell line. (**B**) Effect of the treatments on wound width of MCF-7 cell line. (**C**) Effect of the treatments on wound width of MDA-MB-231 cell line. (**D**) Effect of the treatments on wound width of 4T1 cell line. C: control; FEO: frankincense essential oil; FEO-CSNPs: frankincense essential oil loaded on chitosan nanoparticles; CSNPs: chitosan nanoparticles.

Changes in wound width were measured at time intervals, which reflect the treatment effect at (0 min, 24 h, and 48 h) on the four different cell lines. Figure 9A–D represents the different migratory profiles of C-166, MCF-7, MDA-MB-231, and 4T1 cell lines during the wound healing study. The results showed that there was a non-significant decrease in the wound width of all of the treatments in the C-166 cell line compared to the control after 24 h and 48 h.

On the other hand, the FEO and FEO-CSNPs showed a non-significant decrease, and CSNPs showed a non-significant increase in the wound width in the MCF-7 cell line compared to the control after 24 h. In comparison, after 48 h, FEO showed a significant increase, and FEO-CSNPs and CSNPs showed a non-significant decrease in the wound width compared to the control.

In the MDA-MB-231 cell line, the FEO showed a significant decrease, and the FEO-CSNPs and CSNPs showed a non-significant decrease in wound width compared to the control after 24 h. In comparison, after 48 h, the FEO showed a significant increase, and the FEO-CSNPs and CSNPs showed a non-significant increase in wound width compared to the control.

In the 4T1 cell line, the FEO killed all the cells after 24 h, while the FEO-CSNPs and CSNPs showed a non-significant increase in wound width compared to the control after 24 h and 48 h.

### 3.7. In Vivo Experimental Studies

#### 3.7.1. Body Weight and Tumor Volume

**Figure 10:**
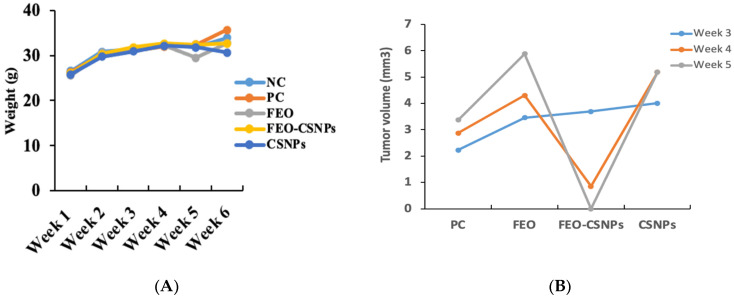
(**A**) Body weight changes in animals over six weeks following the induction of breast tumors. All treatment groups, including negative control (NC), positive control (PC), frankincense essential oil (FEO), frankincense essential oil-loaded chitosan nanoparticles (FEO-CSNPs), and chitosan nanoparticles (CSNPs), showed consistent weight gain, indicating no significant systemic toxicity. (**B**) Tumor volume measurements at 3, 4, and 5 weeks post-treatment. The FEO and FEO-CSNP groups demonstrated a reduction in tumor size, with FEO-CSNPs showing the most pronounced tumor suppression by week 5, suggesting enhanced therapeutic efficacy compared to other treatments.

The changes in the body weight of the animals following the induction of breast tumors over six weeks were recorded (Figure 10A). Across all of the groups—negative control (NC), positive control (PC), frankincense essential oil (FEO), frankincense essential oil-loaded chitosan nanoparticles (FEO-CSNPs), and chitosan nanoparticles (CSNPs)—a gradual increase in body weight was observed. The weight gain appears to be relatively consistent across the treatment groups, with no significant deviations that might indicate adverse effects on overall health or systemic toxicity. In contrast, the tumor volume measurements taken at weeks 3, 4, and 5 post-treatment exhibited some differences (Figure 10B). The tumor sizes varied between the groups, with the positive control (PC) showing a noticeable spike in tumor volume by week 3, followed by a sharp decline over the subsequent weeks. The FEO and FEO-CSNP groups displayed smaller tumor volumes throughout the treatment period, suggesting a potential therapeutic effect in reducing tumor growth. Notably, the FEO-CSNP group exhibited the most sustained reduction in tumor volume, particularly by week 5. The chitosan nanoparticle (CSNP) group followed a similar trend, though with less pronounced reductions compared to the FEO and FEO-CSNP groups. These results suggest that both FEO and FEO-CSNP treatments may offer promise in suppressing tumor growth, with the FEO-CSNP formulation showing enhanced efficacy.

#### 3.7.2. Microscopic Study of Mammary Gland

**Figure 11:**
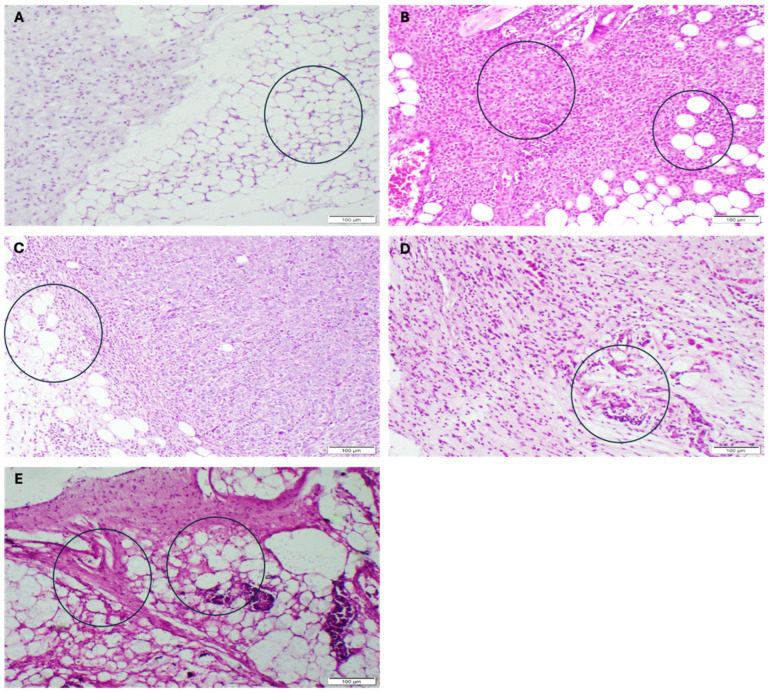
Histopathological analysis of mice mammary glands with hematoxylin and eosin staining, focusing on various treatment groups. (**A**) The mammary gland of negative control shows apparently normal mammary tissue. Adipocytes are clearly visible, and the overall tissue structure is preserved without signs of malignancy. The circle highlights the adipose tissues. (**B**) The mammary gland of positive control shows features of malignancy. The tissue exhibits pleomorphism, hyperchromatic nuclei, and evidence of mitotic activity (circled). (**C**) The mammary gland of FEO shows moderate proliferation of spindle-shaped neoplastic (tumor) cells. These neoplastic cells are associated with leukocytic cells and eosinophil infiltration, which indicates an immune response (shown in the circle). The neoplastic activity is still present but less pronounced compared to the positive control, suggesting that Frankincense oil may be exerting a partial inhibitory effect on tumor growth. (**D**) FEO-CSNP treatment shows a significant reduction in the number of neoplastic cells. There are few spindle-shaped neoplastic cells, and the associated leukocytic infiltration suggests an ongoing immune response. (**E**) CSNP treatment shows severe proliferation of spindle-shaped neoplastic cells with anisocytosis and anisokaryosis (circled).

The histology sections of the breast showed the infiltration of tumor cells. In neoplastic cells, large hyperchromatic nuclei and a relatively small amount of cytoplasm were noted. These neoplastic cells showed criteria of malignancy, including pleomorphic cells, which were hyperchromatic with prominent nuclei and frequent mitosis. The results indicate that the NC group displayed the normal architecture of mice mammary tissue, with stromal connective tissue surrounding the ducts and embedded in adipose tissue (Figure 11A), while the PC group and CSNP group showed pleomorphism, hyperchromatism, and mitotic division of the mice mammary gland (Figure 11B,E). In most areas of the samples treated with the FEO and FEO-CSNPs, the tumor cells were necrotic, and the results showed larger areas of necrosis. Tumor clumps in some areas of tissue were seen (Figure 11C,D).

#### 3.7.3. Stress-Related Parameters

**Figure 12:**
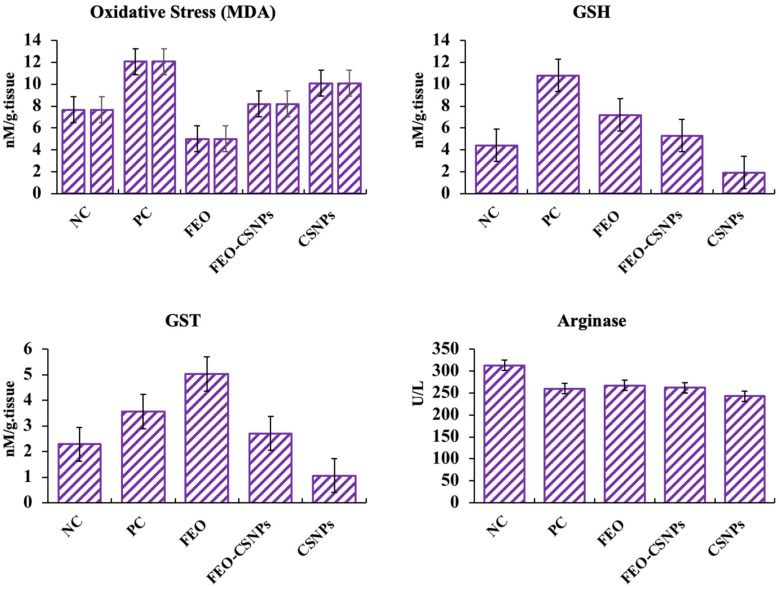
Stress-related parameters in mice treated with different formulations. NC: negative control, PC: positive control, FEO: frankincense essential oil, and CSNP: chitosan nanoparticles. The top-left graph shows baseline stress marker levels, with no significant changes across the NC, FEO, and CSNP groups. The top-right graph highlights a significant increase in stress markers in the PC group, while both FEO and CSNP treatments reduce stress, with CSNP showing the most pronounced effect. The bottom-left graph further demonstrates elevated stress markers in the PC group, while FEO and CSNP effectively mitigate stress responses, particularly in the CSNP-treated group. The bottom-right graph shows stable stress levels across NC, FEO, and CSNP groups, indicating minimal stress induction, while the PC group shows a clear increase. These results suggest that FEO and CSNP, especially CSNP, have potential stress-relieving effects in mice.

Four parameters related to stress responses in mice were measured by comparing the effects of treatments across various groups: negative control (NC), positive control (PC), frankincense essential oil (FEO), and chitosan nanoparticles (CSNPs). Each parameter is represented by a bar graph, showing the levels of stress-related markers under different experimental conditions (Figure 12). NC and PC groups exhibit relatively similar baseline stress levels. At the same time, both the FEO and CSNP treatments maintain stress parameters comparable to the control, with no significant increase in stress markers. This suggests a neutral impact of FEO and CSNP on the baseline stress when compared to the untreated controls.

A clear distinction is visible, where the positive control group shows an elevated response, indicating a heightened stress state. Conversely, the FEO and CSNP treatments reduce this stress parameter significantly compared to the PC group. This suggests a potential stress-relieving effect of both treatments, particularly with CSNPs showing the most notable reduction. The PC group demonstrated a marked increase in stress markers. The FEO treatment effectively moderates the stress response, and CSNPs again show the most prominent reduction in stress markers. This highlights the efficacy of these treatments in mitigating stress responses. The stress levels are relatively consistent across the NC, FEO, and CSNP groups, with a clear elevation in the PC group. These findings reinforce the observation that both the FEO and CSNP treatments appear to alleviate stress, with CSNPs exhibiting the most pronounced effect in reducing stress markers across the measured parameters. This suggests the potential therapeutic use of FEO and CSNP in stress management.

### 3.8. Gene Expression Analysis

To further investigate the efficiency of the FEO, FEO-CSNPs, and CSNPs on the C-166, MCF-7, MDA-MB-231, and 4T1 cells and Balb/c mice mammary glands, the expression of the PIK3CA, CCND1, HER2, STAT3, WNT1, KRAS, TP53, CDH1, and PTEN genes was profiled.

**Figure 13:**
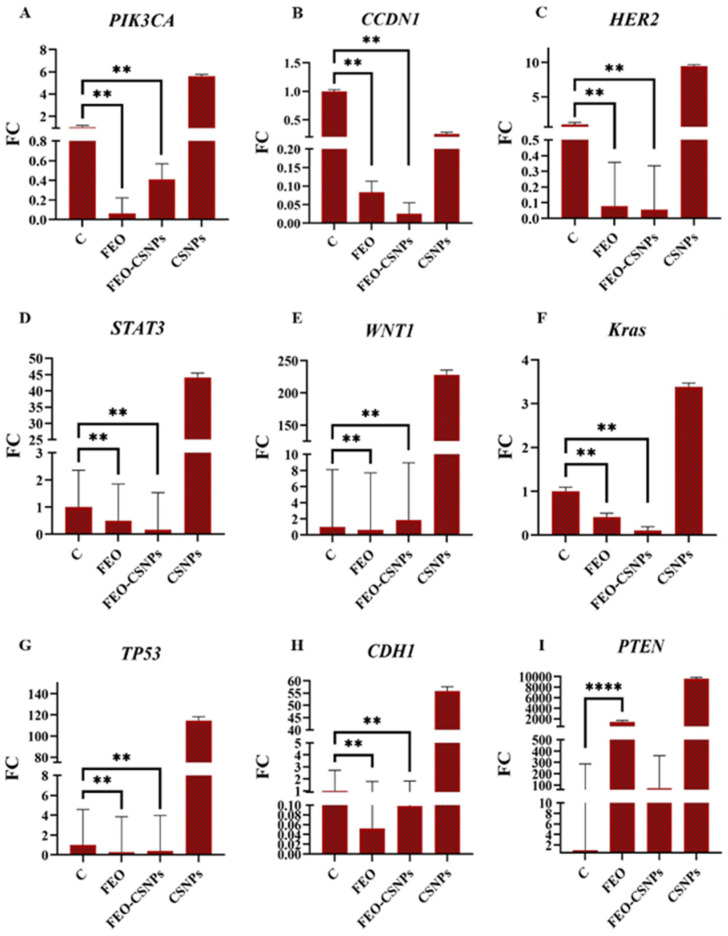
The expression of genes related to cell cycle regulation and tumor suppression, including PIK3CA, CCND1, HER2, STAT3, WNT1, Kras, TP53, CDH1, and PTEN, in C-166 cells treated with FEO, FEO-CSNPs, and CSNPs was assessed by qPCR (**A**–**I**). The fold change (FC) in gene expression compared to the control (**C**) group is displayed, revealing significant modulation of these genes, especially after treatment with FEO-CSNPs, as indicated by statistical significance (** *p* < 0.01, **** *p* < 0.0001). Treatments with FEO-CSNPs and CSNPs showed distinct profiles in regulating oncogenes and tumor suppressor genes, suggesting potential therapeutic benefits.

In the C-166 cell line, the results indicate that the FEO-CSNP treatment had a strong impact on gene expression, particularly in key regulators of cell proliferation and survival, such as PIK3CA, CCND1, HER2, and STAT3. Notably, PIK3CA and HER2 were downregulated by FEO-CSNPs, suggesting an inhibitory effect on pathways critical for cancer cell growth. Conversely, tumor suppressor genes such as TP53 and PTEN were significantly upregulated, which might contribute to enhanced apoptosis and reduced cell proliferation. These findings highlight the potential of FEO-CSNPs as a promising therapeutic strategy in cancer treatment, particularly for targeting the dysregulated molecular pathways in tumor cells (Figure 13).

**Figure 14:**
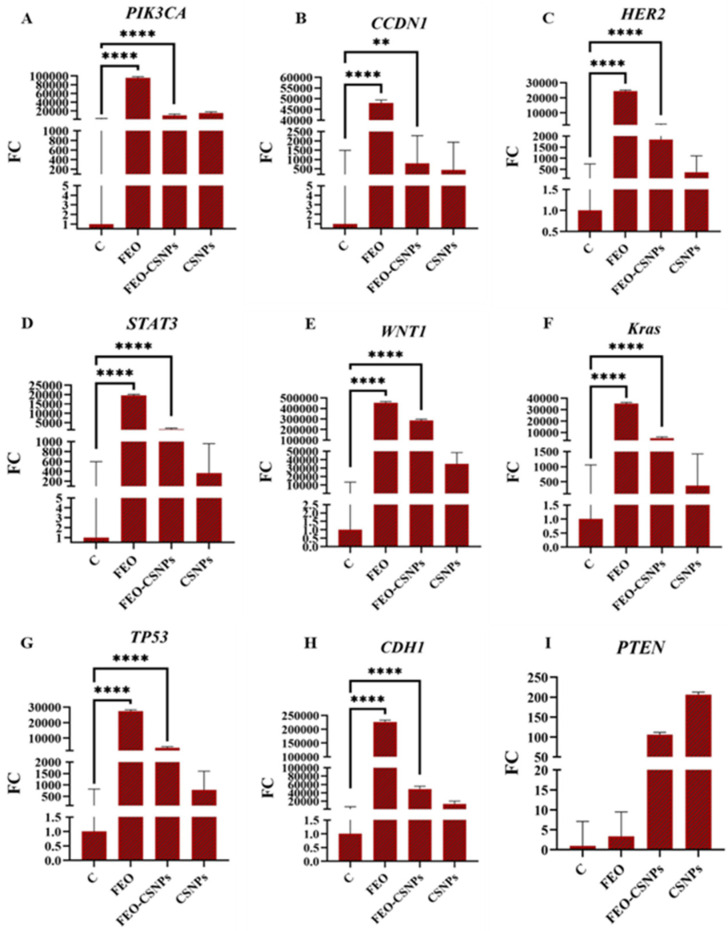
Gene expression analysis of MCF-7 cells treated with FEO, FEO-loaded chitosan nanoparticles (FEO-CSNPs), and chitosan nanoparticles (CSNPs) for 24 h. Relative expression levels of key oncogenes and tumor suppressor genes, such as PIK3CA (**A**), CCND1 (**B**), HER2 (**C**), STAT3 (**D**), WNT1 (**E**), KRAS (**F**), TP53 (**G**), CDH1 (**H**), and PTEN (**I**), were assessed using real-time PCR and normalized to the housekeeping gene GAPDH. The data suggest significant modulation of gene expression, with FEO-CSNPs showing the most substantial changes in both oncogene downregulation and tumor suppressor gene upregulation, indicating potential for therapeutic efficacy in breast cancer treatment. *p*-values of <0.01, and <0.0001 are indicated as ** and ****, respectively.

The data demonstrate that FEO and its nanoparticle formulation significantly impact the expression of several critical genes in the MCF-7 cells. The FEO-CSNPs were particularly effective in downregulating oncogenes like PIK3CA, HER2, KRAS, and CCND1 while simultaneously upregulating tumor suppressors such as TP53, CDH1, and PTEN. These alterations suggest a mechanism where FEO-CSNPs halt tumor growth by both inhibiting cell proliferation pathways and enhancing cell cycle arrest and apoptosis. The potent gene regulatory effects seen in the FEO-CSNP treatment point to its promising role as a therapeutic intervention in breast cancer (Figure 14).

**Figure 15:**
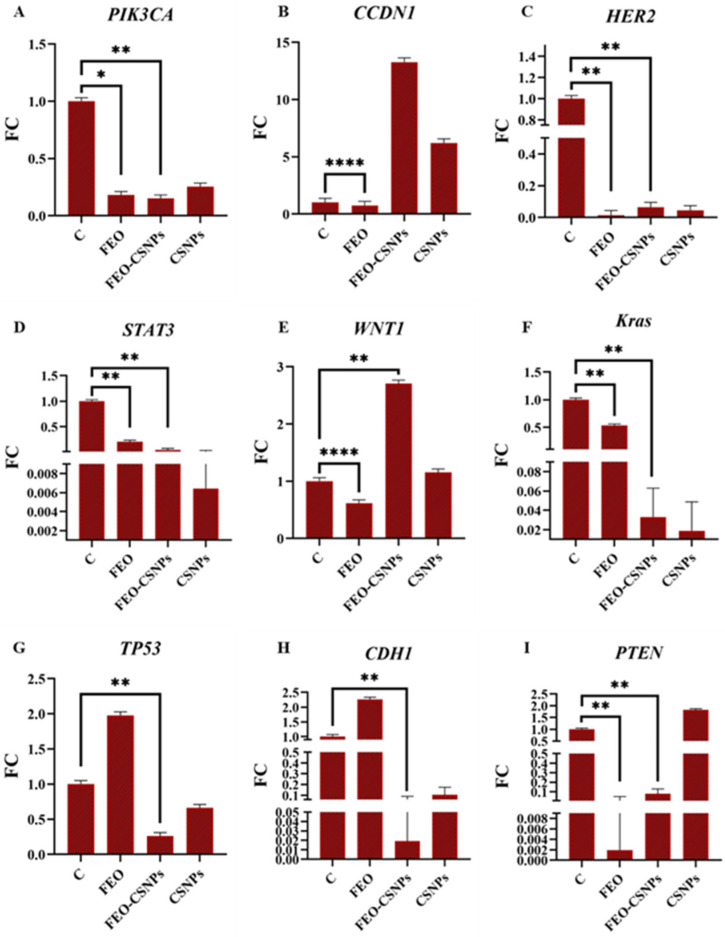
Gene expression profiles of MDA-MB-231 cells treated with FEO, FEO-CSNPs, and CSNPs compared to control. Panels **A** to **I** depict fold changes in the expression of key oncogenes (PIK3CA, CCND1, HER2, STAT3, WNT1, and Kras) and tumor suppressor genes (TP53, CDH1, and PTEN). The treatments resulted in significant modulation of these genes, with FEO-CSNPs showing the most pronounced effects across multiple markers, particularly downregulating oncogenes and upregulating tumor suppressors. *p*-values of <0.05, <0.01, and <0.0001 are indicated as *, ** and ****, respectively. (**A**–**I**) are the genes assessed against various treatments.

In MDA-MB-231 breast cancer cells, oncogenes such as PIK3CA, CCND1, HER2, and WNT1 were significantly downregulated, particularly in the FEO-CSNP treatment group, indicating the suppression of proliferative and metastatic signaling pathways. In contrast, tumor suppressor genes like TP53, CDH1, and PTEN were upregulated, highlighting the potential of FEO-CSNPs in promoting tumor suppression. These findings suggest that FEO-CSNPs could offer an effective therapeutic strategy by targeting multiple pathways involved in breast cancer progression (Figure 15).

**Figure 16:**
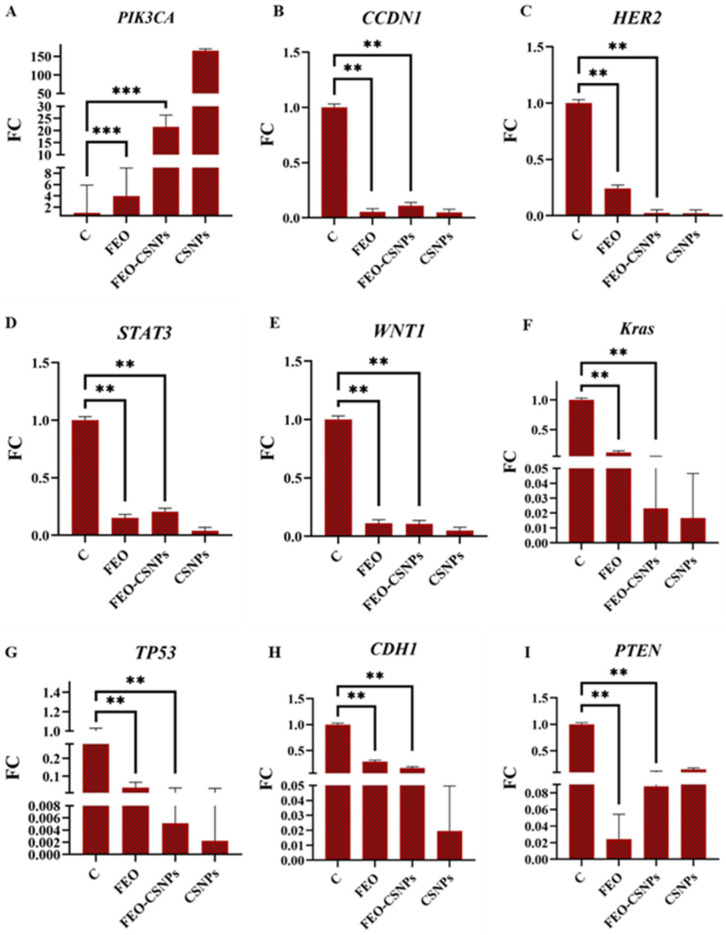
The relative gene expression levels in 4T1 cells treated with FEO, FEO-loaded chitosan nanoparticles (FEO-CSNPs), and chitosan nanoparticles (CSNPs) were analyzed via qPCR. The expression levels of key oncogenes (PIK3CA, CCDN1, HER2, STAT3, WNT1, and KRAS) and tumor suppressor genes (TP53, CDH1, and PTEN) were compared with untreated control cells (C). Significant reductions in oncogene expression and increases in tumor suppressor gene expression were observed in cells treated with FEO and FEO-CSNPs, with FEO-CSNPs showing the most pronounced effects. *p*-values of <0.01, and <0.001 are indicated as ** and ***, respectively. (**A**–**I**) are the genes assessed against various treatments.

The data presented highlight the impact of FEO and FEO-CSNP treatments on the gene expression of both oncogenes and tumor suppressors in 4T1 cells. Notably, the FEO-CSNPs significantly downregulated key oncogenes such as PIK3CA, CCDN1, HER2, and WNT1, while upregulating the tumor suppressors TP53 and CDH1, indicating their potent anti-cancer activity. This effect was more pronounced in the FEO-CSNPs than in the free FEO, suggesting that the nanoparticle formulation enhances the therapeutic efficacy of frankincense essential oil, making it a promising candidate for further development as a cancer treatment (Figure 16).

**Figure 17:**
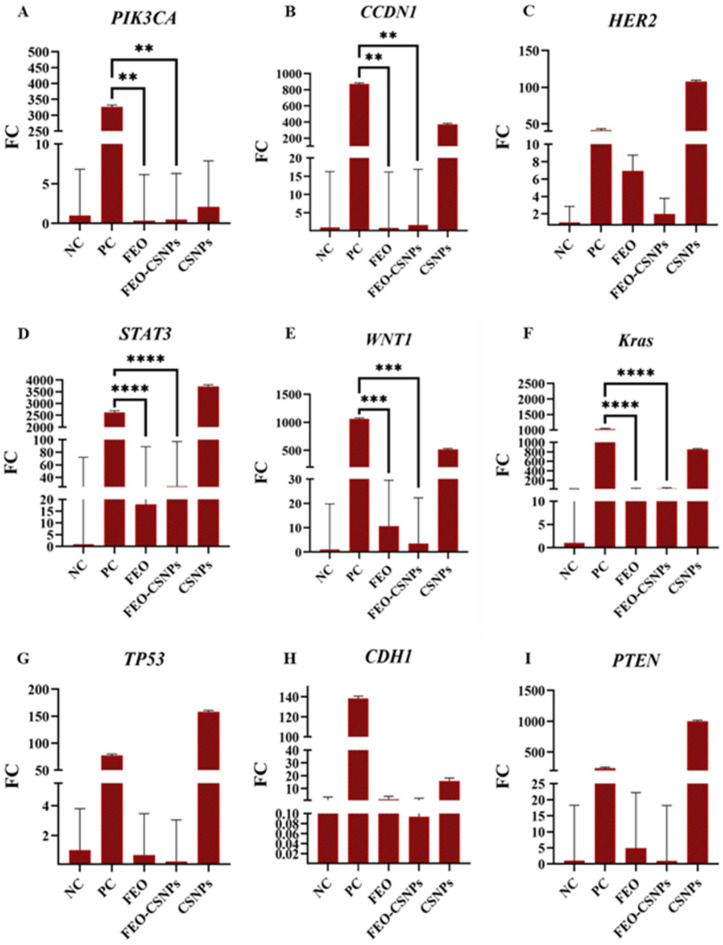
Gene expression analysis in the mammary glands of BALB/c mice after treatment with frankincense essential oil (FEO) and its chitosan nanoparticle-loaded form (FEO-CSNPs). Panels A-I show the relative fold change (FC) of various oncogenes and tumor suppressor genes (PIK3CA, CCDN1, HER2, STAT3, WNT1, KRAS, TP53, CDH1, and PTEN). The results reveal significant modulation of gene expression, with notable increases in tumor suppressor gene activity (TP53, CDH1, and PTEN) in response to FEO-CSNP treatment, compared to the negative control (NC), positive control (PC), and other treatments. *p*-values of <0.01, <0.001, and <0.0001 are indicated as **, ***, and ****, respectively. (**A**–**I**) are the genes assessed against various treatments.

The results indicate that treatment with FEO and FEO-CSNPs influenced the expression of key oncogenes and tumor suppressor genes in the mammary glands of BALB/c mice. FEO-CSNPs led to a more pronounced upregulation of tumor suppressor genes such as TP53, CDH1, and PTEN, suggesting a potential therapeutic role in modulating cancer pathways. Furthermore, the reduction in oncogene expression (e.g., PIK3CA and CCDN1) highlights the effectiveness of the FEO-CSNPs in suppressing tumor growth-related gene activity. This molecular profile supports the hypothesis that the FEO-CSNPs exhibit enhanced anti-cancer effects compared to the FEO or CSNPs alone (Figure 17).

## 4. Discussion

In the Gulf, frankincense essential oils (FEOs) have gained considerable attention for their remarkable biological properties, including their anti-cancer effects, alongside their significant cultural and traditional value [34]. While initial studies have shown promising results, it is crucial to highlight that the current evidence is still in its early stages and requires further validation through comprehensive research. On the other hand, chitosan nanoparticles (CSNPs) have emerged as an innovative tool in drug delivery systems due to their biocompatibility and ability to enhance the therapeutic efficacy of active compounds [35]. The present study focuses on investigating the anti-cancer potential of frankincense essential oil derived from *Boswellia carterii* and its nano-formulation (FEO-CSNPs), particularly in breast cancer cells, to determine their efficacy as novel therapeutic agents.

The GC/MS analysis of *B. carterii* essential oil revealed the presence of several key compounds, each contributing to the oil’s chemical profile and potential biological properties. α-pinene (35.81%) was the main constituent with trans-Verbenol (5.55%), m-Cymene (5.32%) and Verbenone (4.74%). These compounds are predominantly monoterpenes and oxygenated terpenoids, which are characteristic of frankincense essential oil and are known for their diverse pharmacological activities. Our GC-MS profile is in accordance with Camarda, Dayton [36], who reported the presence of α-pinene (37.3%) as a major constituent in *B. carterii* oil. Meanwhile, their results indicated low concentrations of trans-Verbenol (0.5%), Verbenone (0.3%), and m-Cymene (0.3%) in *B. carterii* and *B. rivae* oils. These differences suggest that the composition of *B. carterii* essential oils may be influenced by several factors, such as climatic conditions, seasonal variations, and experimental methods [37].

Numerous studies have highlighted the cytotoxic activity of Boswellia species against cancer cells. Moses, Edwards [38] demonstrated that *Boswellia sacra* combined with gold nanoparticles exhibited significant cytotoxic effects on MDA-MB-231 and MCF-7 breast cancer cells while sparing healthy MCF-10A cells.

The results indicated that the MCF-10A cells were less sensitive to the treatment, particularly at higher concentrations (IC50 = 0.19%). However, both breast cancer cell lines responded similarly to the formulation at lower concentrations (IC50 = 0.13% vs. 0.15%). Similarly, another study showed that frankincense oil induced rapid cell death in J82 bladder cancer cells, completely clearing them from the culture plate within 24 h, while UROtsa cells, a non-tumorigenic line, remained unaffected and retained their typical morphology [39]. Consistent with these findings, our study revealed that the cell viability decreased non-significantly following the treatment with essential oil (FEO), FEO-loaded chitosan nanoparticles (FEO-CSNPs), and chitosan nanoparticles (CSNPs) at concentrations of 10 µg/mL and 100 µg/mL across various cell lines, including C-166, MCF-7, MDA-MB-231, and 4T1 cells. These results underscore the potential selective cytotoxicity of FEO and its nanoparticle formulations.

Fyala and Sultan [40] demonstrated that frankincense essential oil could effectively arrest cancer stem cells in the G1 phase of the cell cycle, particularly in hepatocellular carcinoma lines. In line with this, our study showed that treatments with FEO, FEO-CSNPs, and CSNPs also induced cell cycle arrest across multiple breast cancer cell lines. Specifically, the C-166 and MCF-7 cells were halted at the S phase, MDA-MB-231 cells at the G2/M phase, and 4T1 cells at the G1/S phase. Moreover, we observed that the FEO-CSNPs had a more pronounced effect on apoptosis across all of the cell lines compared to FEO alone, suggesting an enhanced therapeutic potential when the essential oil is encapsulated in nanoparticles. This is consistent with findings by Azzazy, Abdelnaser [41], who reported that encapsulating *Boswellia sacra* oil in PLGA-PCL nanoparticles significantly increased the proportion of necrotic and apoptotic cells compared to that with free oil. These results collectively highlight the promising role of nanoparticle-encapsulated frankincense essential oil in cancer treatment, specifically breast cancer, by enhancing cell cycle arrest and apoptosis. Further studies are warranted to explore this combination in clinical settings.

Alipanah and Zareian [42] reported no statistically significant changes in the body weight between treatment groups receiving *Boswellia serrata* gum resin alcoholic extract at various doses (50, 150, and 250 mg/kg) and a control group across different days (*p* > 0.05). In agreement with these findings, our study revealed a similar trend. There was a non-significant increase in the body weight of the Balb/c mice (from week 1 to week 6) after the treatments compared to control. This suggests that the treatments did not negatively impact the general health of the mice, supporting the safety profile of both the FEO and its nanoparticle formulation.

To evaluate the impact of the treatments used in our study on oxidative stress, we administered frankincense essential oil (FEO) of *Boswellia carterii* at a dose of 6 µL/g and FEO loaded on chitosan nanoparticles (FEO-CSNPs) at a dose of 12 µL/g to female Balb/c mice. Our findings showed a non-significant increase in serum arginase activity levels in the treatment groups compared to the PC group. This aligns with [43], who demonstrated that *C. myrrha* reduced arginase levels following ethanol ingestion in rats, although our study did not replicate this effect. Additionally, Hakkim, Bakshi [16] reported that feeding mice with *Boswellia sacra* oil elevated the hepatic GST levels, reinforcing FEO’s potential role in preserving detoxification processes. Interestingly, our study found a non-significant increase in GST activity in the 6 µL/g FEO treatment group but a significant reduction in the 12 µL/g FEO-CSNP group and a non-significant decrease in the 12 µL/g CSNP group, compared to the PC group.

Similarly, previous research by Yousef [44] demonstrated a significant reduction in liver glutathione (GSH) levels following frankincense administration, consistent with our findings of reduced GSH activity in the FEO, FEO-CSNPs, and CSNPs groups compared to the PC group. Moreover, Elmoslemany, Elzallat [45] and Marefati, Beheshti [46] observed that frankincense extracts reduced malondialdehyde (MDA) levels, further supporting its antioxidant effects. In line with this, our results showed a reduction in MDA levels in both the FEO and CSNP groups, with a significant decrease observed in the FEO-CSNP group compared to in the PC group, suggesting enhanced oxidative stress mitigation by the nano-formulation.

These data highlight the potential of FEO and its nanoparticle formulation as effective agents in mitigating oxidative stress while preserving vital hepatic processes. Further research is warranted to elucidate the underlying mechanisms of these effects.

Breast cancer treatment involves targeting key genes involved in cell proliferation, differentiation, and apoptosis, such as PIK3CA, CCND1, HER2, STAT3, WNT1, and KRAS [47,48,49,50,51,52]. In our study on MDA-MB-231 cells, both frankincense essential oil (FEO) and its chitosan nanoparticle-loaded formulation (FEO-CSNPs) demonstrated significant downregulation of these genes, with FEO showing superior potency in downregulating PIK3CA (*p* = 0.000149), suggesting a potential antagonistic interaction between FEO and the nanocarrier. Previous research, such as that by [53], has similarly reported boswellic acids as suppressing the PI3K pathway, reinforcing our findings.

Additionally, CCND1, overexpressed in more than 50% of mammary tumors [54], was downregulated significantly by both treatments, though the FEO-CSNPs were more potent (*p* = 0.005105). This supports the utility of nano-formulations in enhancing anti-cancer efficacy, consistent with findings by Syrovets, Gschwend [55], who showed boswellic acid derivatives downregulating cyclin D1 in prostate cancer cells.

HER2 overexpression, associated with poor breast cancer prognosis, was also significantly reduced by both the FEO and FEO-CSNPs [56]. The FEO-CSNPs exhibited a more robust HER2 downregulation in MDA-MB-231 cells (*p* = 0.046077), aligning with Al-Balushi, Haque [34] and Salehi, Jamali [31], who highlighted the enhanced efficacy of essential oil nano-formulations over their free counterparts in cancer treatment.

Our findings further showed STAT3 downregulation by both treatments, with the FEO being more potent in reducing its expression (*p* = 0.036708). STAT3, implicated in cancer progression and chemoresistance [57], was similarly targeted by essential oil-based treatments in studies by Thalappil, Butturini [58], validating our results.

Moreover, both the FEO and FEO-CSNPs downregulated WNT1, a gene linked to accelerated tumor growth and poor prognosis [59,60]. Although the FEO showed greater efficacy, the results were consistent with those in [61], where boswellic acid’s inhibition of Wnt signaling in cancer was demonstrated.

Regarding KRAS, the FEO and FEO-CSNPs significantly downregulated its expression in the MDA-MB-231 cells (*p* = 0.045668), further confirming the potential of these treatments in targeting RAS-related pathways, as supported by [62].

Lastly, the tumor suppressor genes TP53, CDH1, and PTEN were significantly upregulated by the FEO alone, with the FEO-CSNPs showing less pronounced effects. The upregulation of TP53 (*p* = 0.0000000007) and CDH1 (*p* = 0.0000002) by FEO aligns with studies by Abd-Rabou and Edris [63] and Takahashi, Sung [64], confirming their essential roles in promoting apoptosis and reducing metastasis in breast cancer. Similarly, PTEN upregulation by FEO underscores the potential of boswellic acids in restoring tumor-suppressive pathways [65].

These results underscore the therapeutic potential of frankincense essential oil, particularly when combined with nanocarriers, in the treatment of aggressive breast cancers. Further clinical studies are warranted to explore these formulations’ full anti-cancer capabilities.

## 5. Conclusions

In this study, we employed both in vivo and in vitro approaches to comprehensively assess the anti-tumor potential of *Boswellia carterii* essential oil (FEO) and its nano-formulation (FEO-CSNPs) against breast cancer. Our results revealed significant suppression of tumor growth in the 4T1 murine breast cancer model, accompanied by notable inhibitory effects on MCF-7, MDA-MB-231, and 4T1 breast cancer cell lines. These findings collectively suggest that both FEO and FEO-CSNPs exhibit strong anti-cancer activity, with enhanced potential when utilizing the nano-formulation. These outcomes highlight the therapeutic promise of FEO, particularly when integrated with nanocarriers, as an innovative strategy for breast cancer treatment. While our study provides compelling preliminary evidence, further research is imperative to explore the precise molecular mechanisms by which FEO exerts its anti-tumor effects. Additional studies are also needed to optimize the delivery of FEO-CSNPs and validate their therapeutic efficacy and safety in clinical settings. Future research should also focus on addressing potential drug resistance, long-term efficacy, and pharmacokinetics to fully harness FEO’s potential as a novel breast cancer treatment. These insights may lay the groundwork for developing novel, targeted therapeutic strategies that integrate natural bioactive with advanced drug delivery technologies.

## Figures and Tables

**Table 1:** Designed primer sequences using NCBI’s blast tool for real-time analysis.

Gene Name	Forward	Reverse
*GAPDH*	TAGGCGCTCACTGTTCTCTC	GCCCAATACGACCAAATCCG
*PIK3CA*	ACCCGATGCGGTTAGAGC	TGATGGTCGTGGAGGCATTG
*CCND1*	GACCCCGCACGATTTCATTG	TGGAGGGCGGATTGGAAATG
*HER2*	AGTGAGCAAGTGATGTCCTGA	ACCCCCATACTTGTCCCTTGA
*STAT3*	GGAAGAATCCAACAACGGCA	TGGCAAGGAGTGGGTATCA
*WNT1*	CCCCTTTGTCCTGCGTTTTC	CATTTCTGCTGGTTCCCCCA
*KRAS*	AGGGACTAGGGCAGTTTGGA	AATGTCTTGGCACACCACCA
*TP53*	GAGACCTGTGGGAAGCGAAA	CTGGCATTCTGGGAGCTTCA
*CDH1*	CTGATGCTGATGCCCCCAA	AGCTGTGAGGATGCCAGTTT
*PTEN*	CTCAGCCGTTACCTGTGTGT	AGGTTTCCTCTGGTCCTGGT

**Table 2:** List of the chemical constituents of *B. carterii* frankincense essential oil.

Peak No	Retention Time	Compound Name	Molecular Formula	Molecular Weight	RetIndex	Peak Area (%)
1	5.599	Methanol, (1,4-dihydrophenyl)-	C7H10O	110	988	0.14
2	6.638	Sabinene	C10H16	136	897	1.85
3	6.912	α-thujene	C10H16	136	902	2.85
4	7.246	α-pinene	C10H16	136	948	35.81
5	7.535	Camphene	C10H16	136	943	2.31
6	7.692	2,4(10)-Thujadiene	C10H14	134	879	2.95
7	8.075	1,3,3,4-Tetramethyl-2-oxabicyclo [2.2.0]hexane	C9H16O	140	892	0.08
8	8.328	β-pinene	C10H16	136	943	2.36
9	8.777	4,7-Methano-1H-indene, 2,4,5,6,7,7a-hexahydro-	C10H14	134	891	0.87
10	8.955	2-Butanone, 4-cyclopentylidene-	C9H14O	138	1118	0.13
11	9.176	1,4-Cyclohexadiene, 3-ethenyl-1,2-dimethyl-	C10H14	134	1013	0.53
12	9.373	Benzene, 1-methoxy-2-methyl-	C8H10O	122	983	0.89
13	9.813	m-Cymene	C10H14	134	1042	5.32
14	9.932	Limonene	C10H16	136	1018	2.96
15	10.215	p-Cymene	C10H14	134	1042	0.17
16	10.535	2-Cyclohexen-1-ol, 1-methyl-4-(1-methylethenyl)-, trans-	C10H16O	152	1140	0.10
17	10.835	γ-terpinene	C10H16	136	998	0.39
18	11.250	3-Cyclohexene-1-methanol, 2-hydroxy-.alpha.,.alpha.,4-trimethyl-	C10H18O2	170	1331	0.42
19	11.595	(1R)-cis-Verbenol	C10H16O	152	1136	0.42
20	11.802	Benzene, 1-methyl-4-(1-methylethenyl)-	C10H12	132	1073	1.44
21	12.150	(+)-Nerolidol	C15H26O	222	1564	0.67
22	12.331	2-Cyclohexen-1-ol, 1-methyl-4-(1-methylethenyl)-, trans-	C10H16O	152	1140	0.53
23	12.575	Fenchol	C10H18O	154	1138	1.55
24	12.935	α-Campholenal	C10H16O	152	1155	0.76
25	13.389	Sabinol	C10H16O	152	1131	6.47
26	13.599	trans-Verbenol	C10H16O	152	1136	5.55
27	14.015	E-pinocamphone	C10H16O	152	1109	1.12
28	14.254	α-Phellandren-8-ol	C10H16O	152	1125	2.86
29	14.537	terpinen-4-ol	C10H18O	154	1137	1.38
30	14.854	p-Cymen-8-ol	C10H14O	150	1197	1.65
31	14.984	α-Terpineol	C10H18O	154	1143	1.15
32	15.111	Myrtenal	C10H14O	150	1136	2.74
33	15.567	Verbenone	C10H14O	150	1119	4.74
34	15.829	trans-Carveol	C10H16O	152	1206	1.57
35	16.175	Carveol	C10H16O	152	1206	0.27
36	16.413	2-Methyl-7-exo-vinylbicyclo[4.2.0]oct-1(2)-ene	C11H16	148	1112	0.40
37	16.535	Carvone	C10H14O	150	1190	0.34
38	16.755	Myrtenyl formate	C11H16O2	180	1312	0.45
39	17.190	3,5-Dimethoxytoluene	C9H12O2	152	1172	0.50
40	17.435	1-Cyclohexene-1-carboxaldehyde, 4-(1-methylethyl)-	C10H16O	152	1175	0.08
41	17.726	Bornyl acetate	C12H20O2	196	1277	1.12
42	17.975	(+)-cis-Verbenol, acetate	C12H18O2	194	1276	0.13
43	18.135	Shisool acetate	C12H20O2	196	1374	0.10
44	33.941	n-Hexadecanoic acid	C16H32O2	256	1968	0.20
45	34.075	(R,1E,5E,9E)-1,5,9-Trimethyl-12-(prop-1-en-2-yl)cyclotetradeca-1,5,9-triene	C20H32	272	2121	0.31
46	37.398	Oleic Acid	C18H34O2	282	2175	0.58
47	37.615	Cycloheptane, 4-methylene-1-methyl-2-(2-methyl-1-propen-1-yl)-1-vinyl-	C15H24	204	1475	0.14
48	37.855	Incensole isomer	C20H34O2	306	2303	0.68

## Data Availability

All generated data are enclosed within this publication.

## Supplementary Materials

The mass spectra of the identified compounds in *B. Carterii* essential oil can be downloaded at https://www.mdpi.com/article/10.3390/pharmaceutics17040426/s1.

## Abbreviations

AKBA: acetyl-11-keto-β-boswellic acid; *B. carterii*: *Boswellia carterii*; BO: *Boswellia* oil; BO/PLGA-PCL NPs: *Boswellia* oil/Poly(lactic-co-glycolic acid)–Poly(ε-caprolactone) nanoparticles; *C. myrrha*: *Commiphora myrrha*; CDNB: 1-chloro-2,4-dinitrobenzene; CSNPs: chitosan nanoparticles; DLS: dynamic light scattering; DTNB: 5,5′-dithio-bis(2-nitrobenzoic acid); E-cad: e-cadherin; EE: entrapment efficiency; EPR: enhanced permeability and retention; FBS: fetal bovine serum; FEO: frankincense essential oil; FEO-CSNPs: frankincense essential oil loaded on chitosan nanoparticles; FITC: fluorescein isothiocyanate; FT-IR: Fourier transform infrared; GC/MS: gas chromatography/mass spectrometry; GSH: glutathione; GST: glutathione-S-transferase; IACUC: Institutional Animal Care and Use Committee; LC: loading capacity; LPO: lipid peroxidation; MDA: malonaldehyde; PCL: poly(ε-caprolactone); PCR: polymerase chain reaction; PDI: polydispersity index; PI3K: phosphoinositide-3-kinase; PLGA: polylactide-coglycolide; PLGA-PCL NPs: Poly(lactic-co-glycolic acid)–Poly(ε-caprolactone) nanoparticles; SRB: sulforhodamine B; TCA: trichloroacetic acid; TEM: transmission electron microscopy; TPP: tripolyphosphate; TRIS: tris(hydroxymethyl)aminomethane.
