# Supplementary material for: Anti-Tumor Potential of Frankincense Essential Oil and Its Nano-Formulation in Breast Cancer: An In Vivo and In Vitro Study
Source: Pharmaceutics. 2025 Mar 27;17(4):426. doi: 10.3390/pharmaceutics17040426 (PMC12030047; doi:10.3390/pharmaceutics17040426)
Supplement: Supplementary file 1 [file pharmaceutics-17-00426-s001.zip › pharmaceutics-3512906-supplementary.pdf]

Table S1: Mass spectra of the identified constituents in *Boswellia carterii* essential oil

Peak No	Compound Name	Mass spectra
1	Methanol, (1,4-dihydrophenyl)-	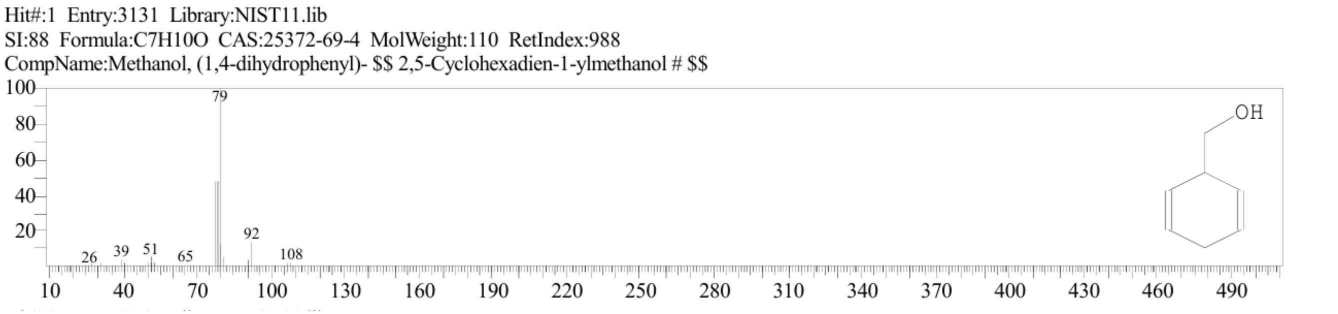
2	Sabinene	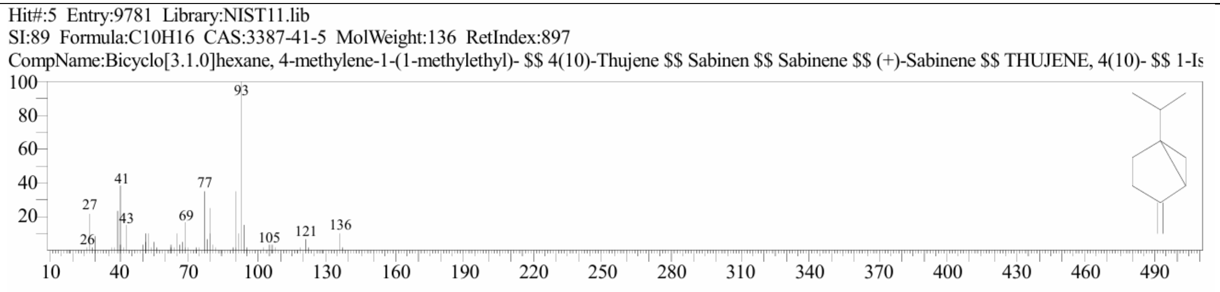

3	α-thujene	Hit#:1 Entry:9791 Library:NIST11.lib<br>SI:97 Formula:C10H16 CAS:2867-05-2 MolWeight:136 RetIndex:902<br>CompName:Bicyclo[3.1.0]hex-2-ene, 2-methyl-5-(1-methylethyl)- $$ 3-Thujene $$ .alpha.-Thujene $$ Origanene $$ 5-Isopropyl-2-methylbicyclo[3.1.0]hex<br>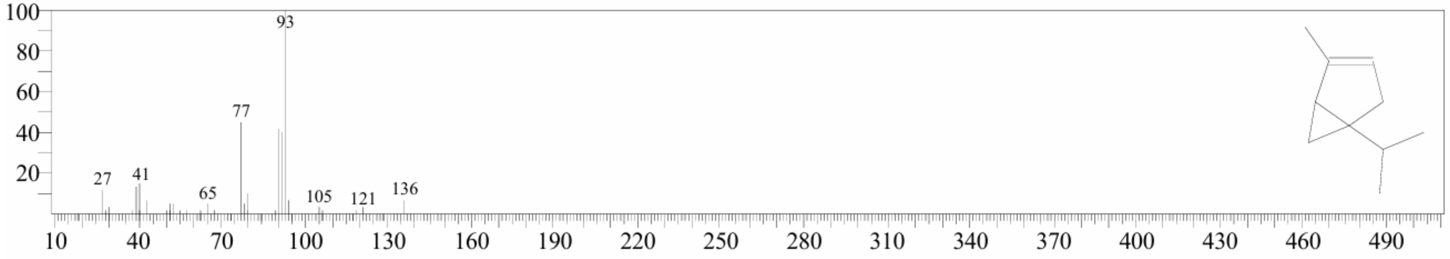
4	α-pinene	Hit#:1 Entry:6669 Library:NIST11s.lib<br>SI:97 Formula:C10H16 CAS:80-56-8 MolWeight:136 RetIndex:948<br>CompName:.alpha.-Pinene $$ Bicyclo[3.1.1]hept-2-ene, 2,6,6-trimethyl- $$ 2-Pinene $$ 2,6,6-Trimethylbicyclo[3.1.1]hept-2-ene $$ Pinene, .alpha. $$ 2,6,6<br>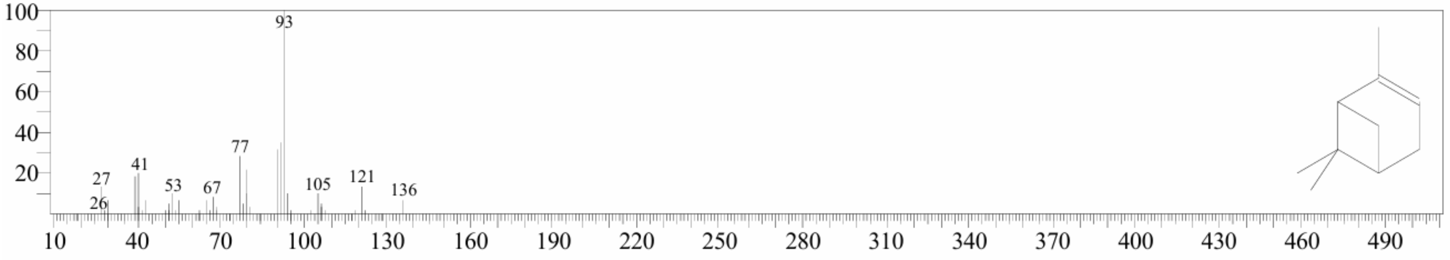

5	Camphene	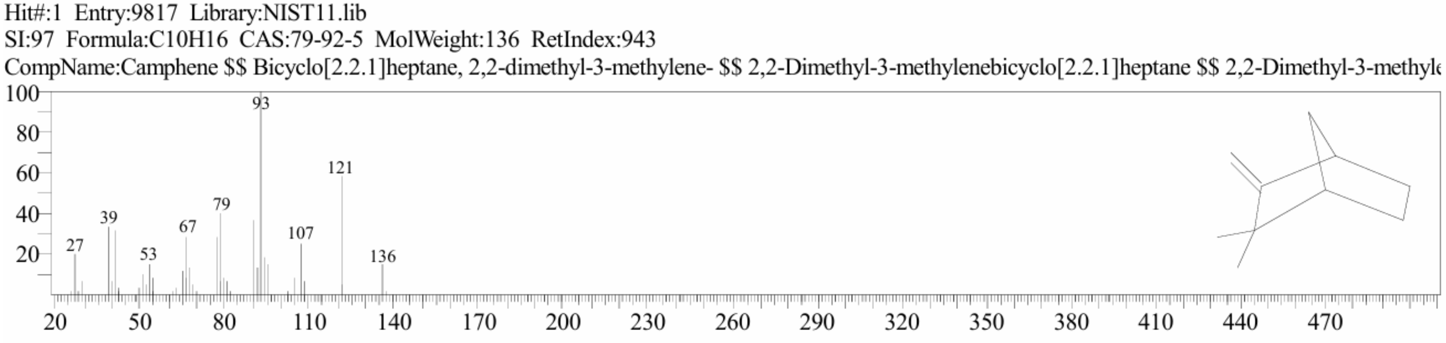
6	2,4(10)-<br><br>Thujadiene	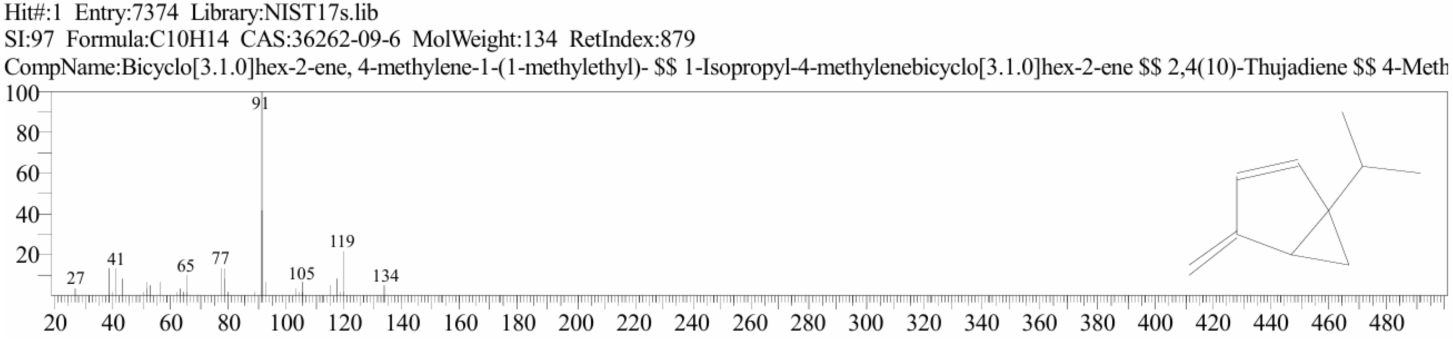

7	1,3,3,4-Tetramethyl-2-oxabicyclo[2.2.0]hexane	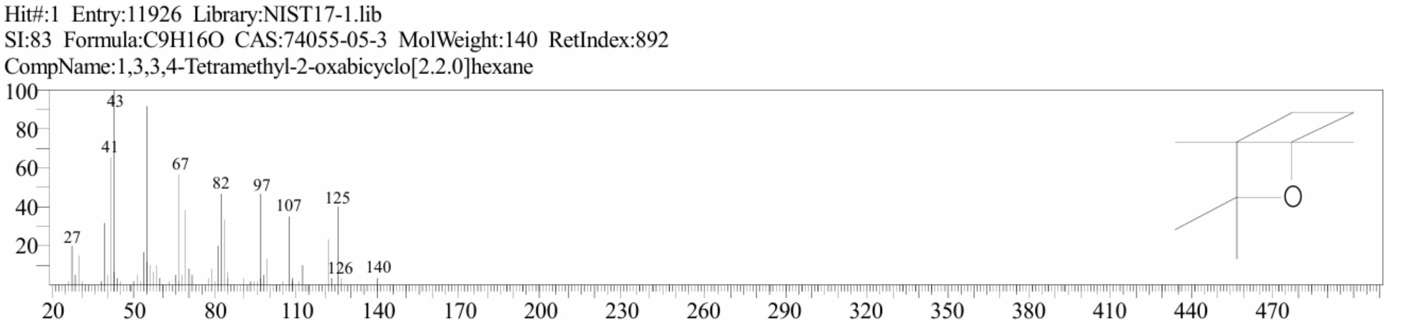
8	β-pinene	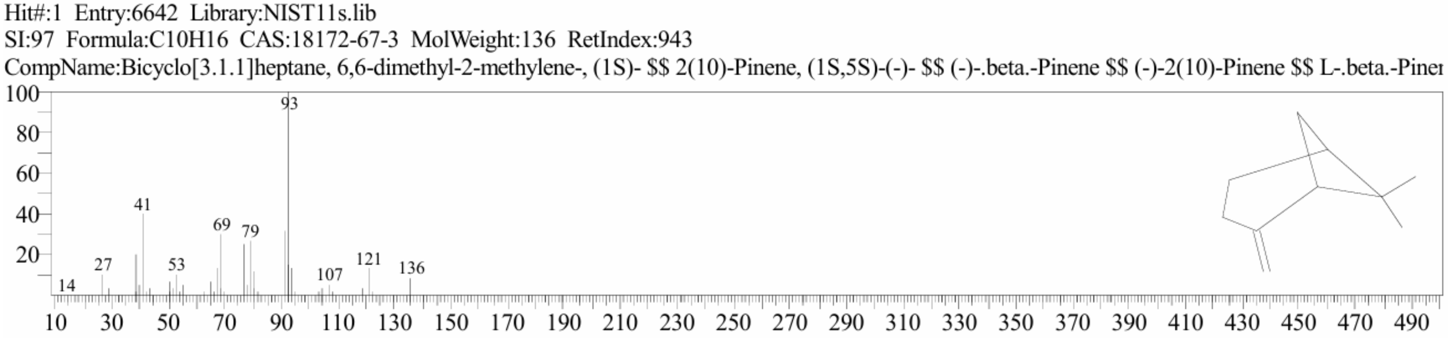

9	4,7-Methano-1H-indene, 2,4,5,6,7,7a-hexahydro-	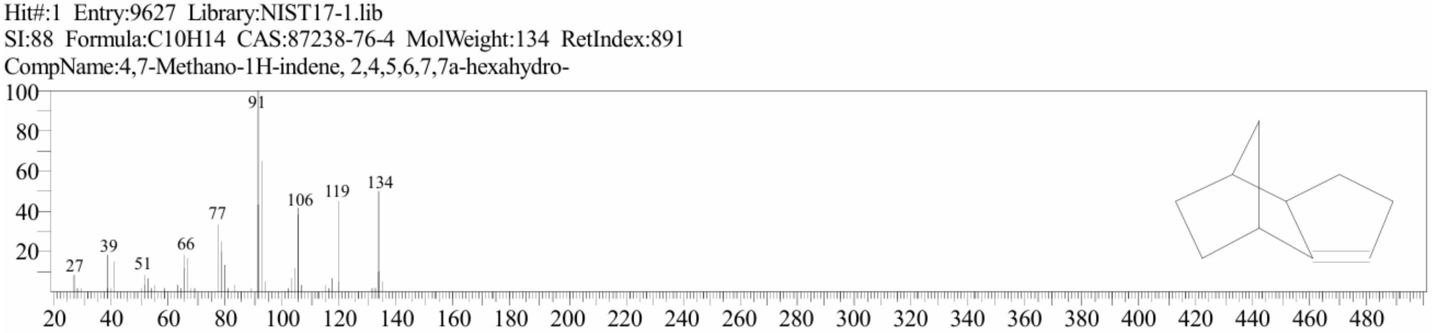
10	2-Butanone, 4-cyclopentylidene-	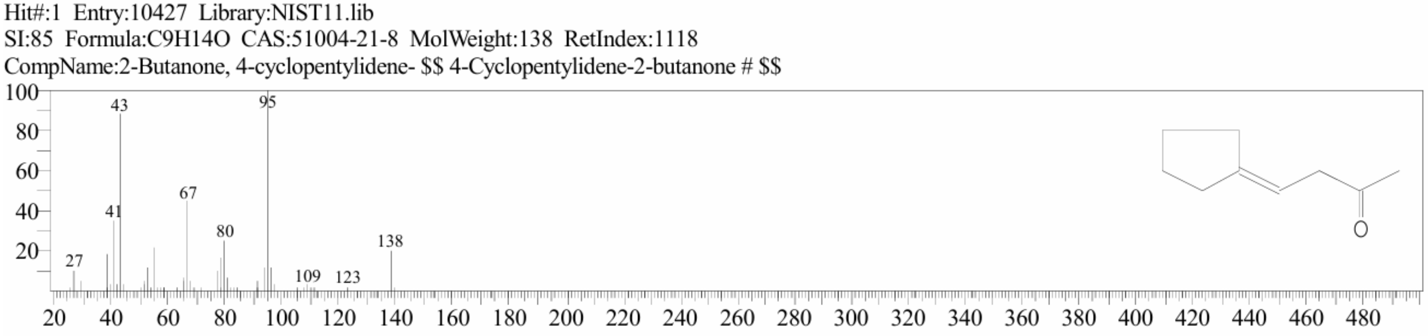

11	1,4- Cyclohexadiene, 3-ethenyl-1,2- dimethyl-	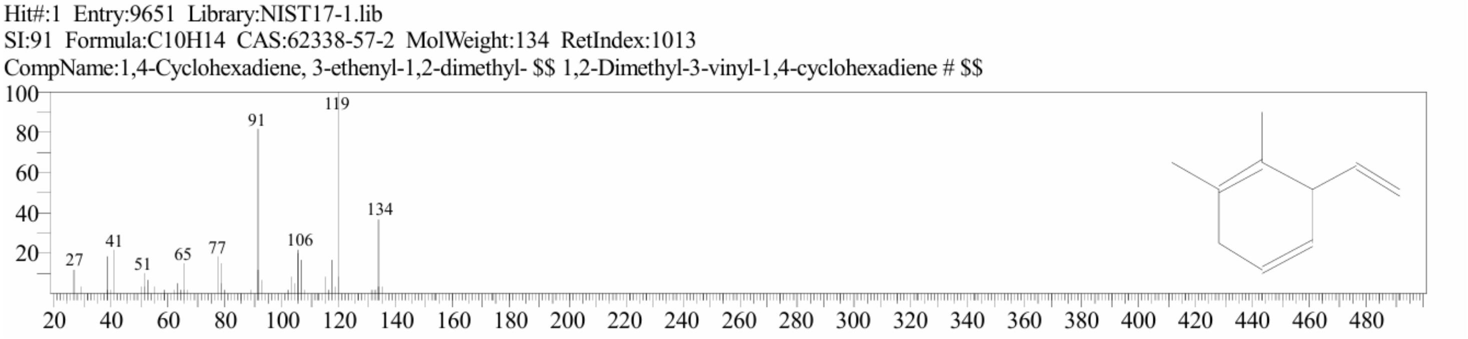
12	Benzene, 1- methoxy-2- methyl-	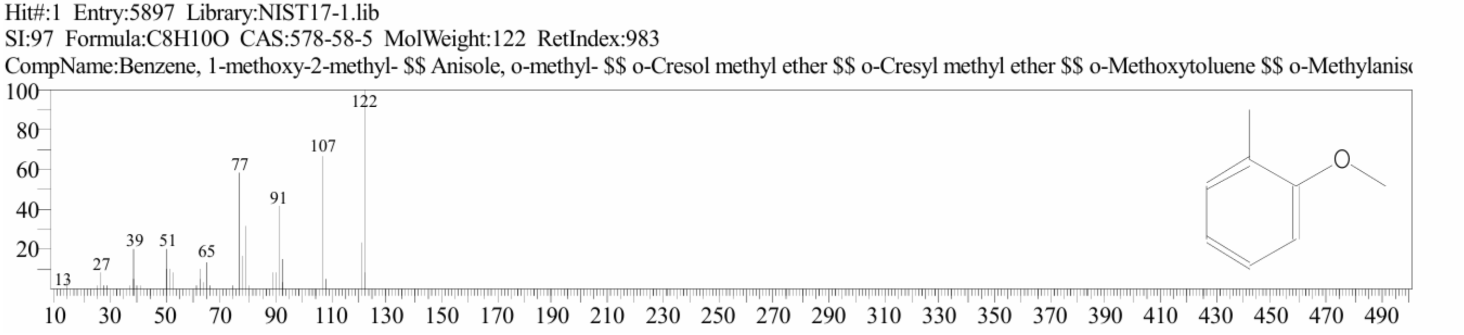

13	m-Cymene	Hit#:2 Entry:6230 Library:NIST11s.lib<br>SI:96 Formula:C10H14 CAS:535-77-3 MolWeight:134 RetIndex:1042<br>CompName:Benzene, 1-methyl-3-(1-methylethyl)- $$ m-Cymene $$ .beta.-Cymene $$ m-Cymol $$ m-Isopropyltoluene $$ m-Methylisopropylbenzene $$ 1·<br>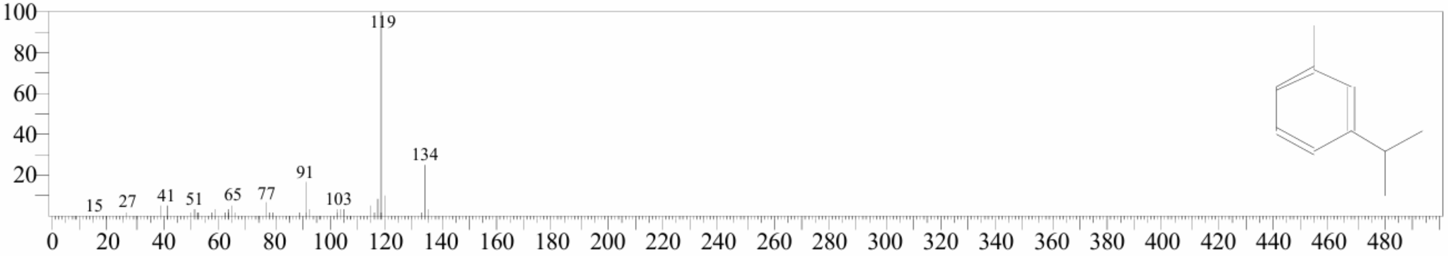
14	Limonene	Hit#:2 Entry:6614 Library:NIST11s.lib<br>SI:93 Formula:C10H16 CAS:138-86-3 MolWeight:136 RetIndex:1018<br>CompName:Limonene $$ Cyclohexene, 1-methyl-4-(1-methylethenyl)- $$ p-Mentha-1,8-diene $$ .alpha.-Limonene $$ Cajeputen $$ Cajeputene $$ Cinen $<br>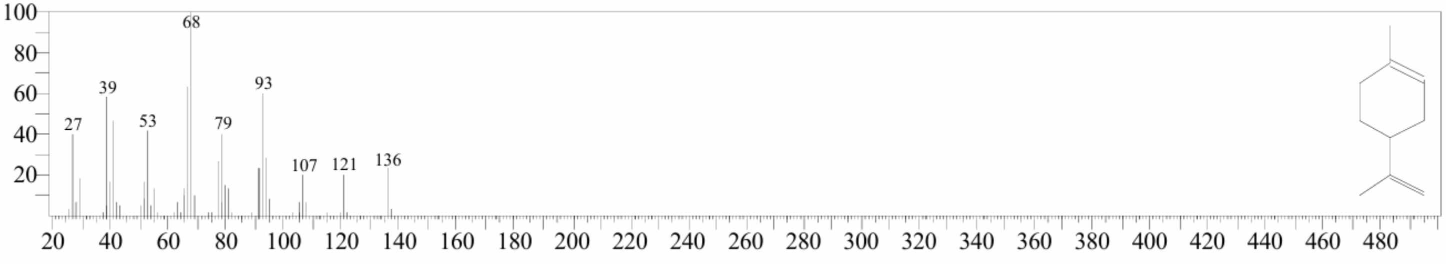

15	p-Cymene	Hit#:1 Entry:7405 Library:NIST17s.lib<br>SI:95 Formula:C10H14 CAS:99-87-6 MolWeight:134 RetIndex:1042<br>CompName:p-Cymene $$ Benzene, 1-methyl-4-(1-methylethyl)- $$ p-Cimene $$ p-Cymol $$ p-Isopropyltoluene $$ p-Methylisopropylbenzene $$ Campho<br>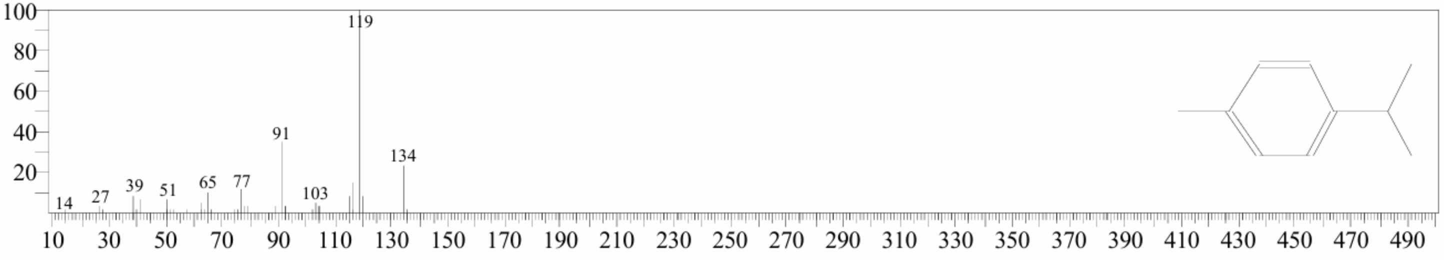
16	2-Cyclohexen-1-ol, 1-methyl-4-(1-methylethenyl)-, trans-	Hit#:1 Entry:16327 Library:NIST11.lib<br>SI:80 Formula:C10H16O CAS:7212-40-0 MolWeight:152 RetIndex:1140<br>CompName:2-Cyclohexen-1-ol, 1-methyl-4-(1-methylethenyl)-, trans- $$ 1R,4R-p-Mentha-2,8-dien-1-ol $$ (E)-p-2,8-Menthadien-1-ol $$ (E)-p-Menth-2,8-<br>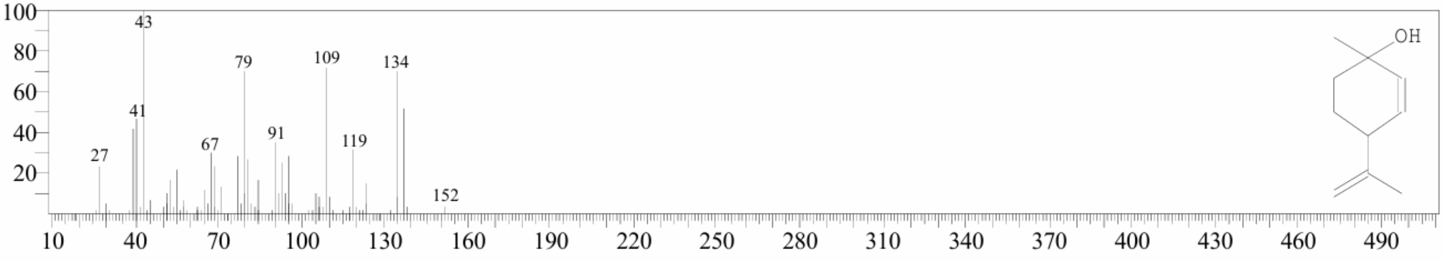

17	γ-terpinene	Hit#:1 Entry:6656 Library:NIST11s.lib<br>SI:97 Formula:C10H16 CAS:99-85-4 MolWeight:136 RetIndex:998<br>CompName:.gamma.-Terpinene $$ 1,4-Cyclohexadiene, 1-methyl-4-(1-methylethyl)- $$ .gamma.-Terpinen $$ p-Mentha-1,4-diene $$ Crithmene $$ Moslene<br>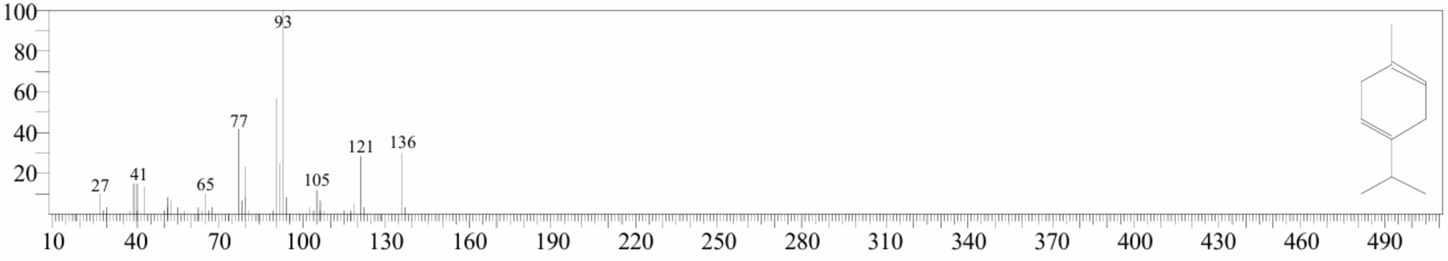
18	3-Cyclohexene-1-methanol, 2-hydroxy-.alpha.,.alpha.,4-trimethyl-	Hit#:1 Entry:15456 Library:NIST17s.lib<br>SI:83 Formula:C10H18O2 CAS:6252-34-2 MolWeight:170 RetIndex:1331<br>CompName:3-Cyclohexene-1-methanol, 2-hydroxy-.alpha.,.alpha.,4-trimethyl- $$ 6-(1-Hydroxy-1-methylethyl)-3-methyl-2-cyclohexen-1-ol # $$<br>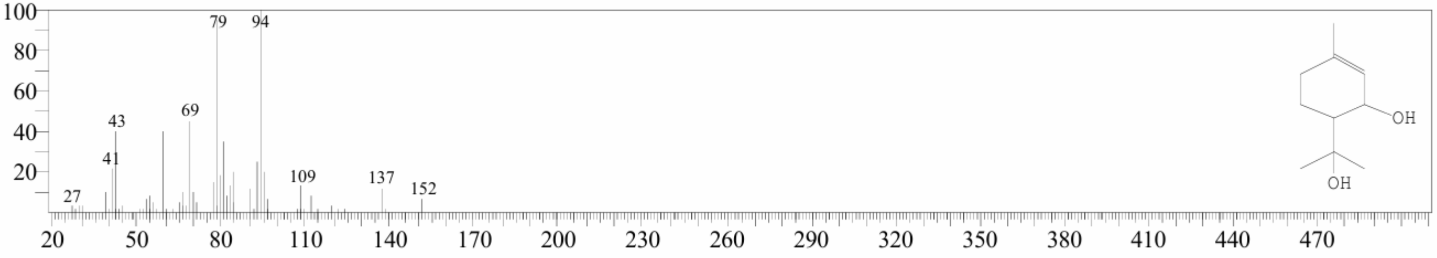

19	(1R)-cis-<br>Verbenol	Hit#:1 Entry:17564 Library:NIST17-1.lib<br>SI:88 Formula:C10H16O CAS:13040-03-4 MolWeight:152 RetIndex:1136<br>CompName:(1R)-cis-Verbenol $$ Bicyclo[3.1.1]hept-3-en-2-ol, 4,6,6-trimethyl-, [1R-(1.alpha.,2.beta.,5.alpha.)]- $$ 2-Pinen-4-ol, (1R,4R,5R)-(+)- $$ (+)-ci<br>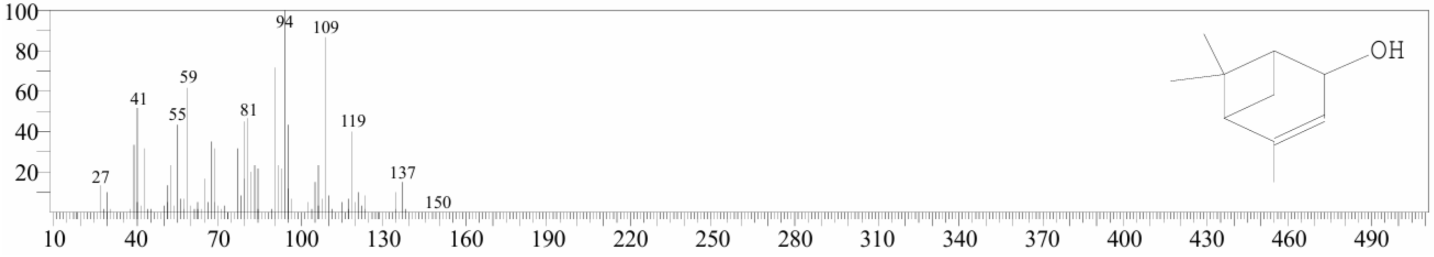
20	Benzene, 1-<br>methyl-4-(1-<br>methylethenyl)-	Hit#:1 Entry:8601 Library:NIST11.lib<br>SI:89 Formula:C10H12 CAS:1195-32-0 MolWeight:132 RetIndex:1073<br>CompName:Benzene, 1-methyl-4-(1-methylethenyl)- $$ p-Cymenene $$ Styrene, p,.alpha.-dimethyl- $$ .alpha.,p-Dimethylstyrene $$ .alpha.,4-Dimethylstyr<br>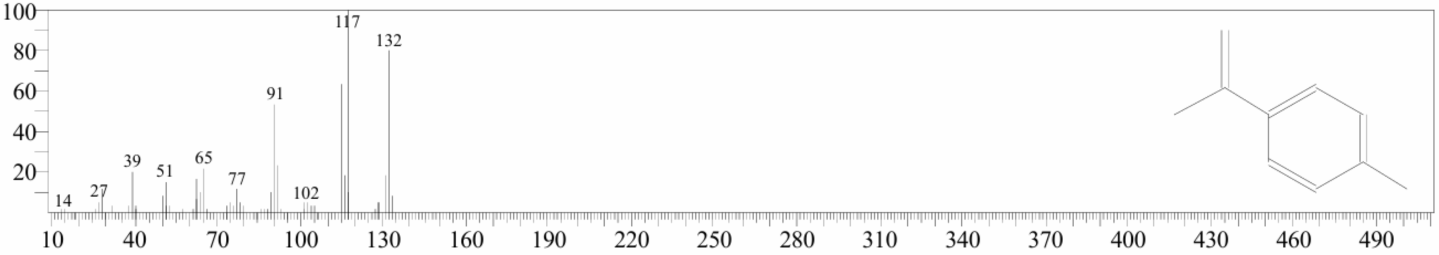

21	(+)-Nerolidol	Hit#:1 Entry:20274 Library:NIST11s.lib<br>SI:87 Formula:C15H26O CAS:142-50-7 MolWeight:222 RetIndex:1564<br>CompName:1,6,10-Dodecatrien-3-ol, 3,7,11-trimethyl-, [S-(Z)]- $$ 1,6,10-Dodecatrien-3-ol, 3,7,11-trimethyl-, (Z)-(S)-(+)- $$ (+)-Nerolidol $$ D-nerolidol<br>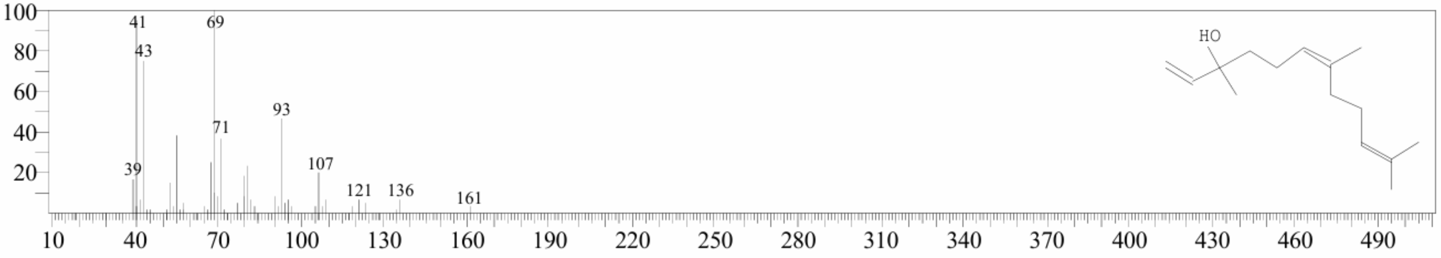
22	2-Cyclohexen-1-ol, 1-methyl-4-(1-methylethenyl)-, trans-	Hit#:1 Entry:9475 Library:NIST11s.lib<br>SI:86 Formula:C10H16O CAS:7212-40-0 MolWeight:152 RetIndex:1140<br>CompName:2-Cyclohexen-1-ol, 1-methyl-4-(1-methylethenyl)-, trans- $$ 1R,4R-p-Mentha-2,8-dien-1-ol $$ (E)-p-2,8-Menthadien-1-ol $$ (E)-p-Menth-2,8-<br>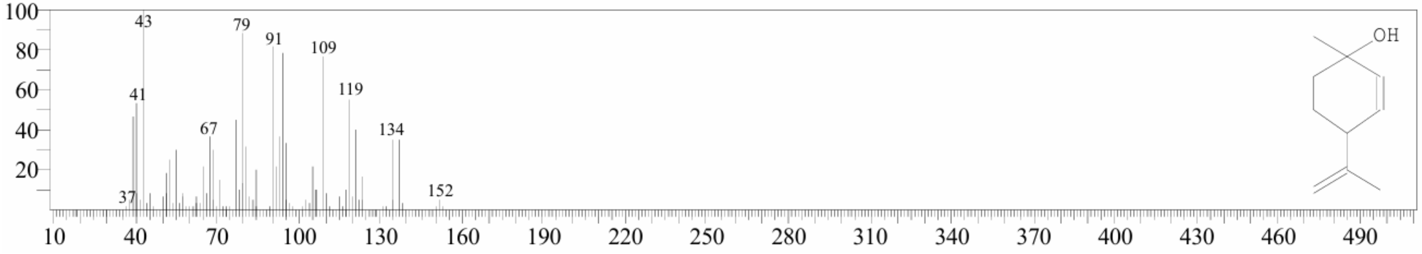

23	Fenchol	Hit#:1 Entry:11993 Library:NIST17s.lib<br>SI:96 Formula:C10H18O CAS:1632-73-1 MolWeight:154 RetIndex:1138<br>CompName:Fenchol $$ Bicyclo[2.2.1]heptan-2-ol, 1,3,3-trimethyl- $$ 2-Norbornanol, 1,3,3-trimethyl- $$ Fenchyl alcohol $$ 1,3,3-Trimethyl-2-norbornano<br>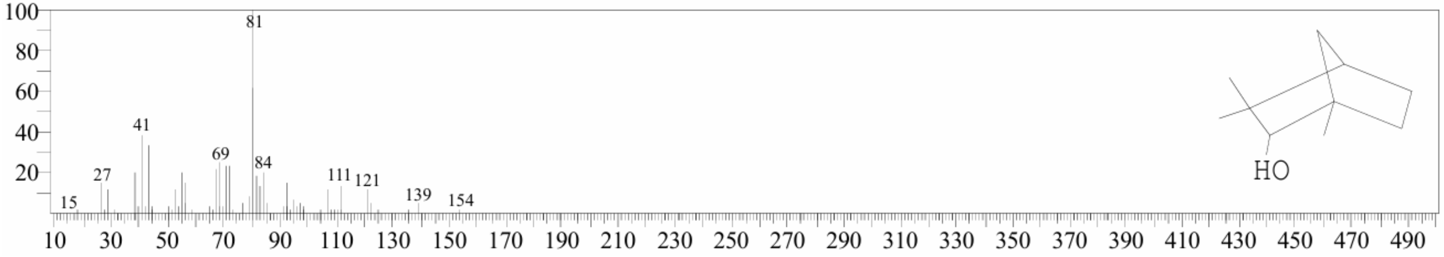
24	α-Campholenal	Hit#:1 Entry:9560 Library:NIST11s.lib<br>SI:93 Formula:C10H16O CAS:4501-58-0 MolWeight:152 RetIndex:1155<br>CompName:.alpha.-Campholenal $$ (R)-.alpha.-Campholene aldehyde $$ 3-Cyclopentene-1-acetaldehyde, 2,2,3-trimethyl- $$ .alpha.-Compholene aldehyde<br>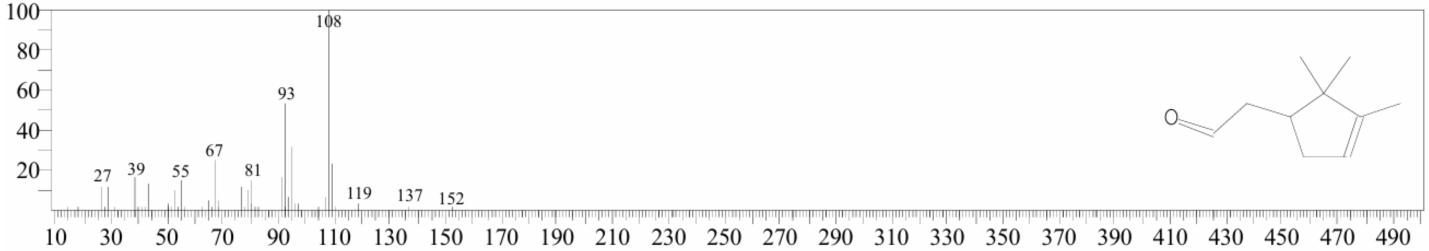

25	Sabinol	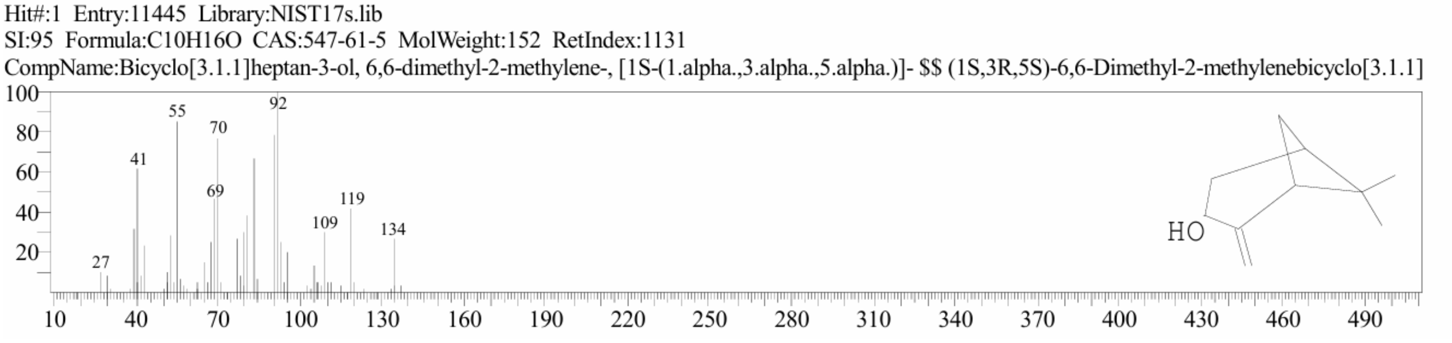
26	trans-Verbenol	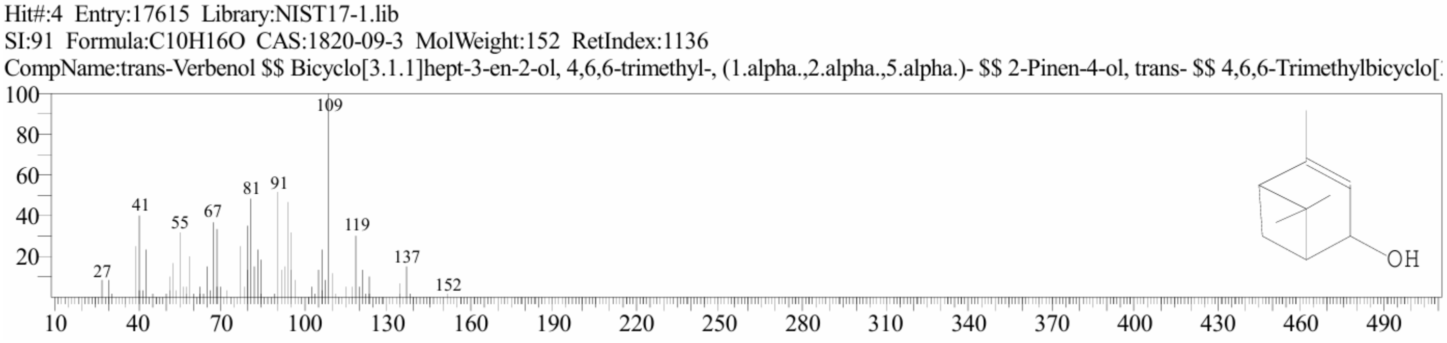

27	E-pinocamphone	Hit#:1 Entry:11439 Library:NIST17s.lib<br>SI:97 Formula:C10H16O CAS:547-60-4 MolWeight:152 RetIndex:1109<br>CompName:Bicyclo[3.1.1]heptan-3-one, 2,6,6-trimethyl-, (1.alpha.,2.alpha.,5.alpha.)- $$ trans-3-Pinanone $$ (E)-Pinocamphone $$ trans-Pinocamphone $$<br>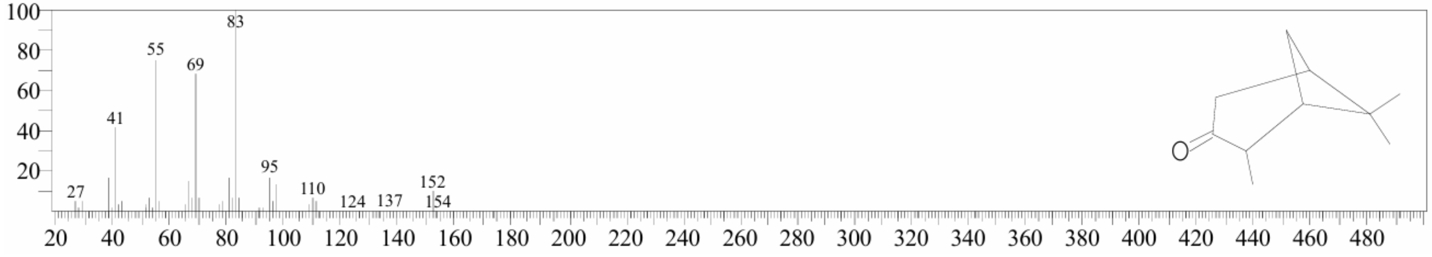
28	α-Phellandren-8-<br><br>ol	Hit#:1 Entry:17447 Library:NIST17-1.lib<br>SI:96 Formula:C10H16O CAS:1686-20-0 MolWeight:152 RetIndex:1125<br>CompName:p-Mentha-1,5-dien-8-ol $$ 2,4-Cyclohexadiene-1-methanol, .alpha.,.alpha.,4-trimethyl- $$ .alpha.-Phellandren-8-ol $$ 2-(4-Methyl-2,4-cyclohe<br>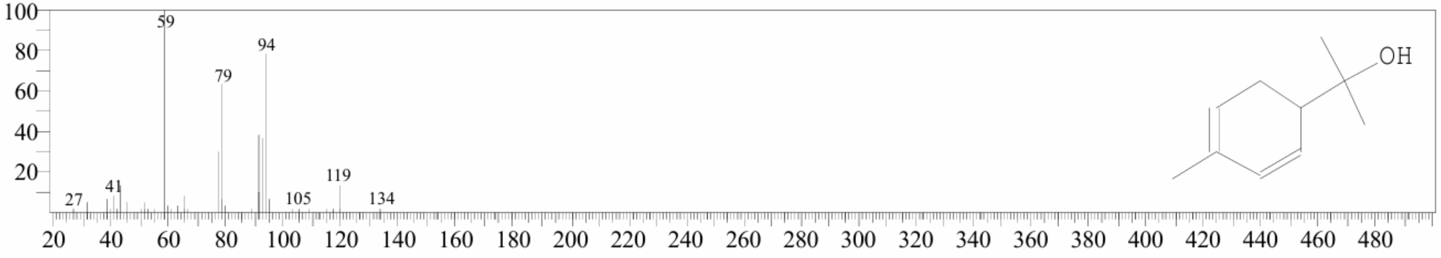

29	terpinen-4-ol	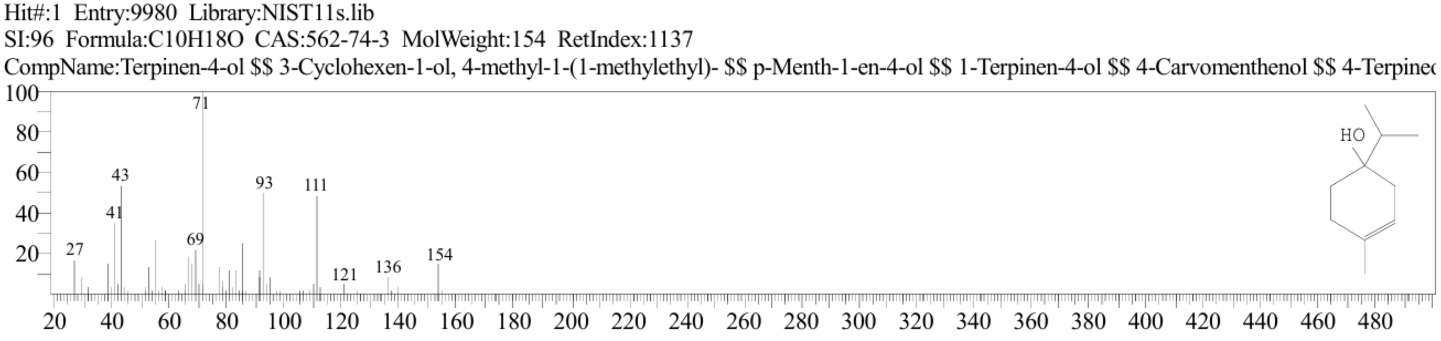
30	p-Cymen-8-ol	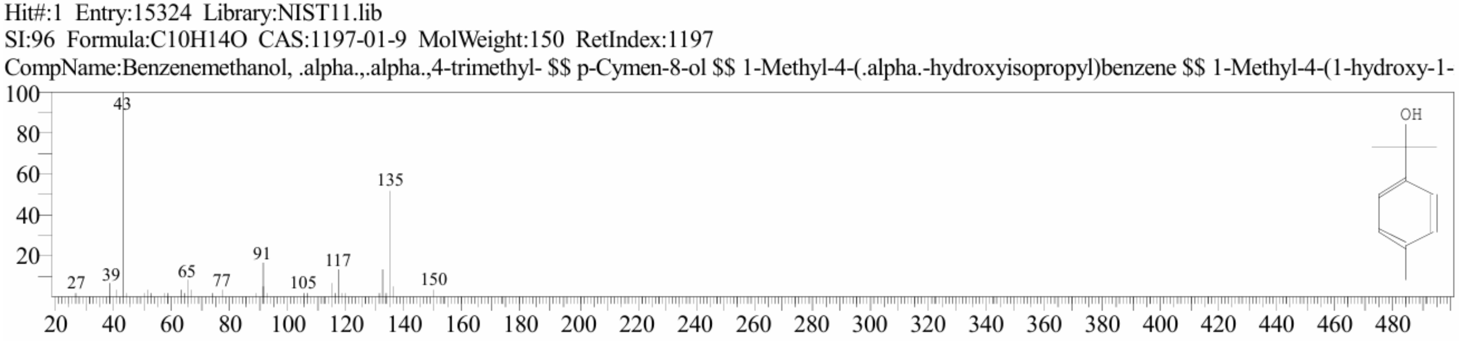

31	α-Terpineol	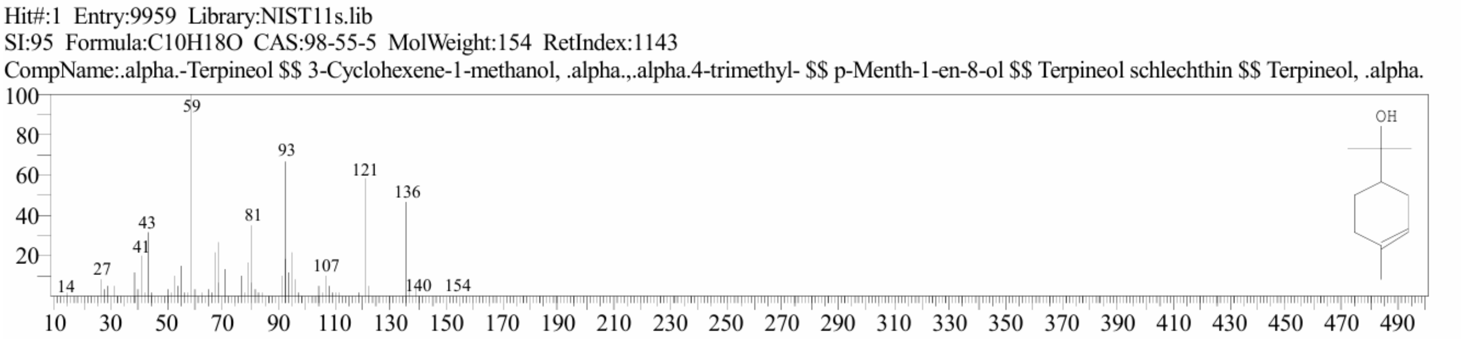
32	Myrtenal	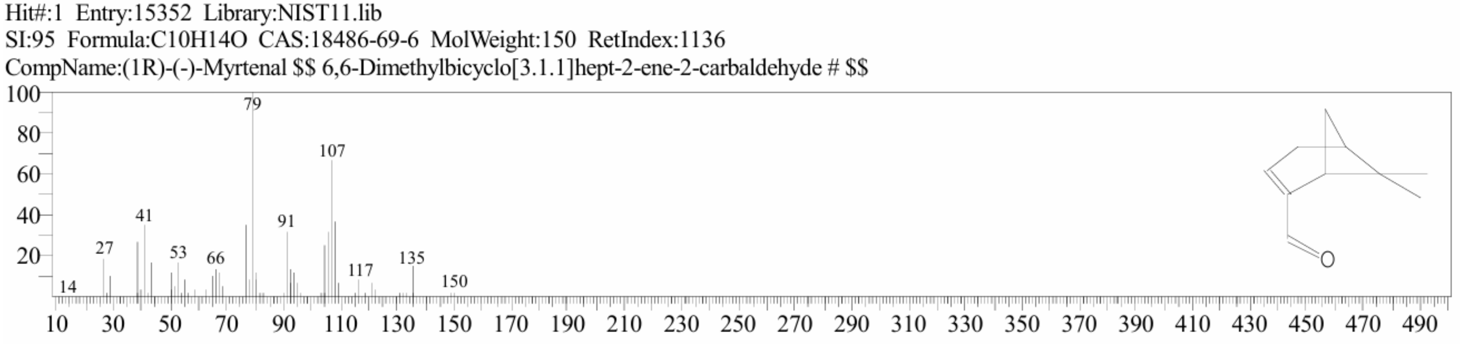

33	Verbenone	Hit#:2 Entry:15419 Library:NIST11.lib<br>SI:95 Formula:C10H14O CAS:80-57-9 MolWeight:150 RetIndex:1119<br>CompName:Bicyclo[3.1.1]hept-3-en-2-one, 4,6,6-trimethyl- $$ 2-Pinen-4-one $$ Berbenone $$ Verbenone $$ 4,6,6-Trimethylbicyclo[3.1.1]hept-3-en-2-one<br>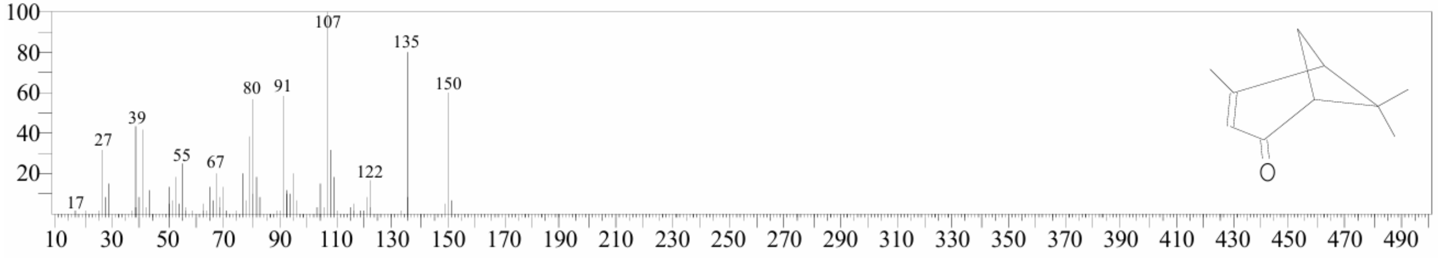
34	trans-Carveol	Hit#:2 Entry:11483 Library:NIST17s.lib<br>SI:96 Formula:C10H16O CAS:1197-07-5 MolWeight:152 RetIndex:1206<br>CompName:trans-Carveol $$ 2-Cyclohexen-1-ol, 2-methyl-5-(1-methylethenyl)-, trans- $$ p-Mentha-6,8-dien-2-ol, trans- $$ (E)-Carveol $$ t-Carveol $$ tra<br>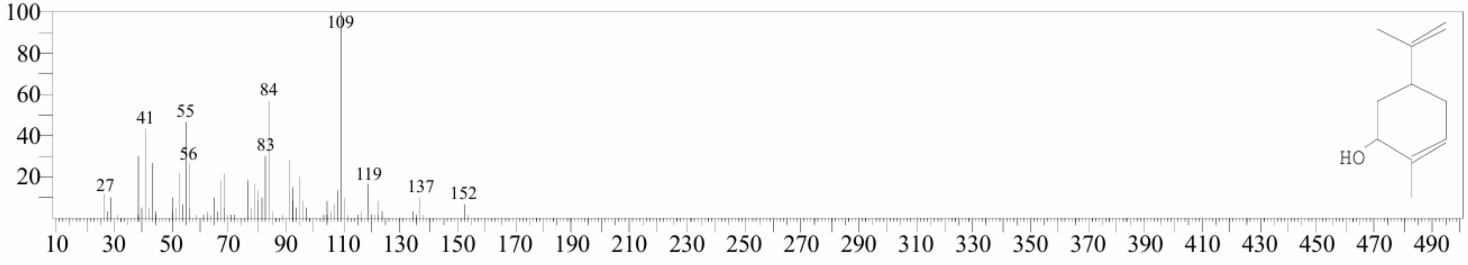

35	Carveol	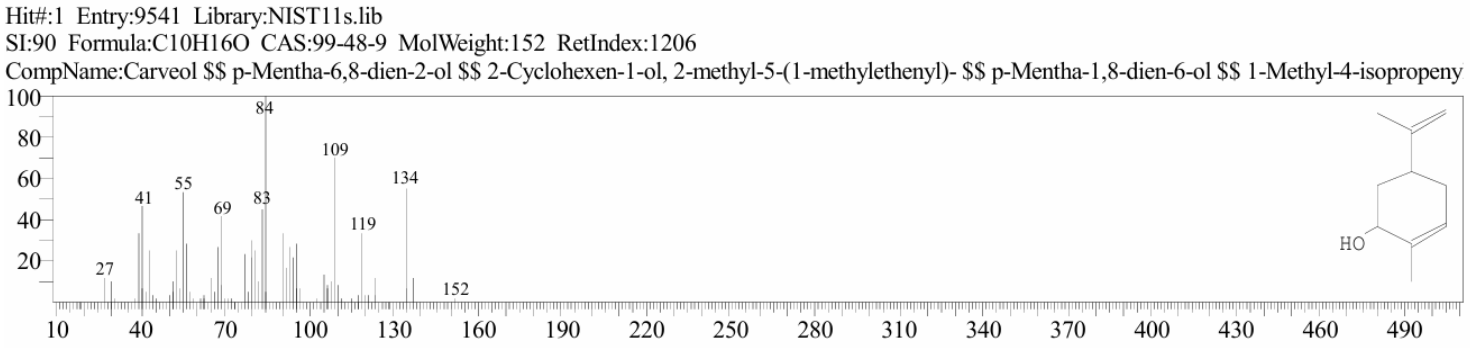
36	2-Methyl-7-exo-vinylbicyclo[4.2.0]oct-1(2)-ene	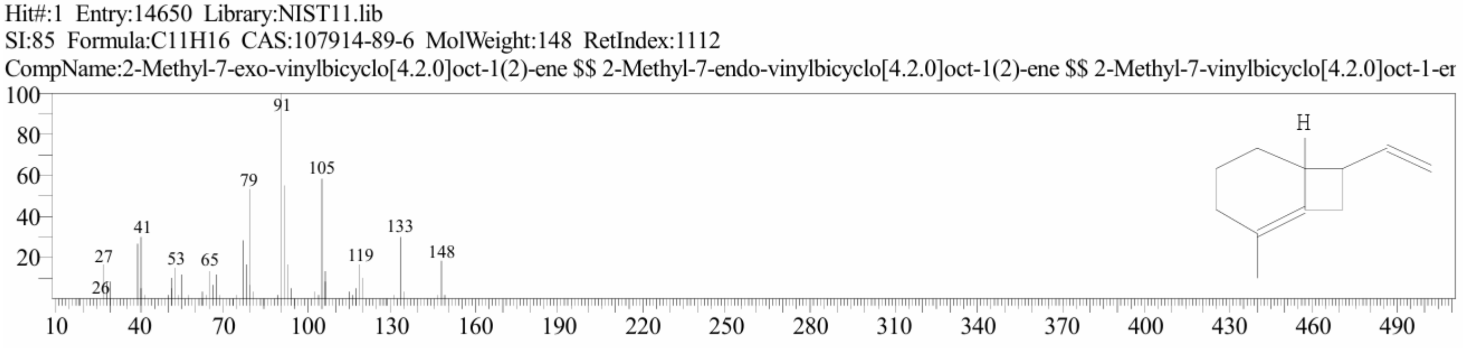

37	Carvone	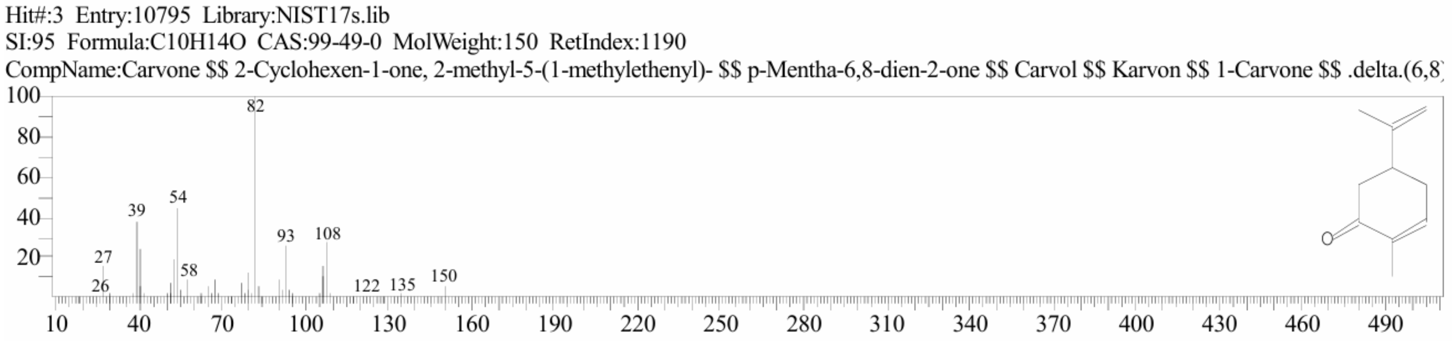
38	Myrtenyl formate	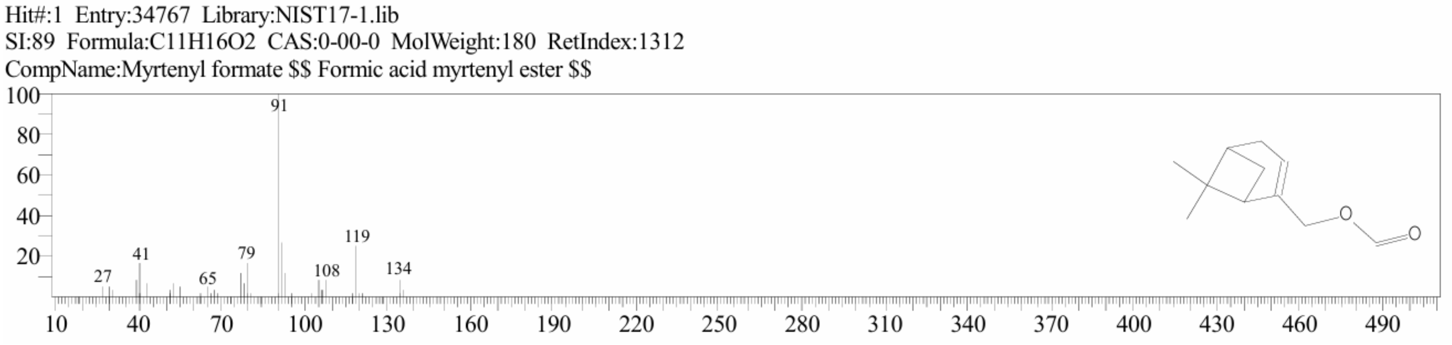

39	3,5-Dimethoxytoluene	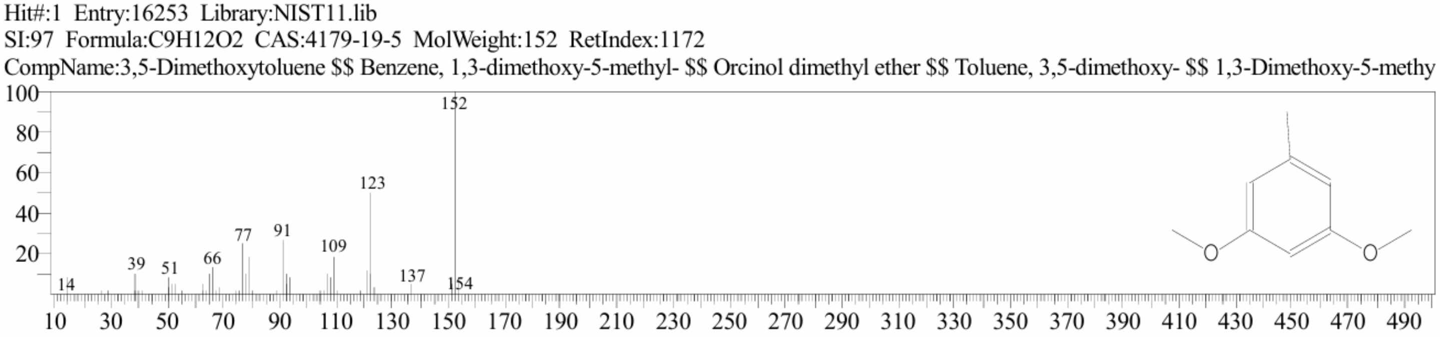
40	1-Cyclohexene-1-carboxaldehyde, 4-(1-methylethyl)-	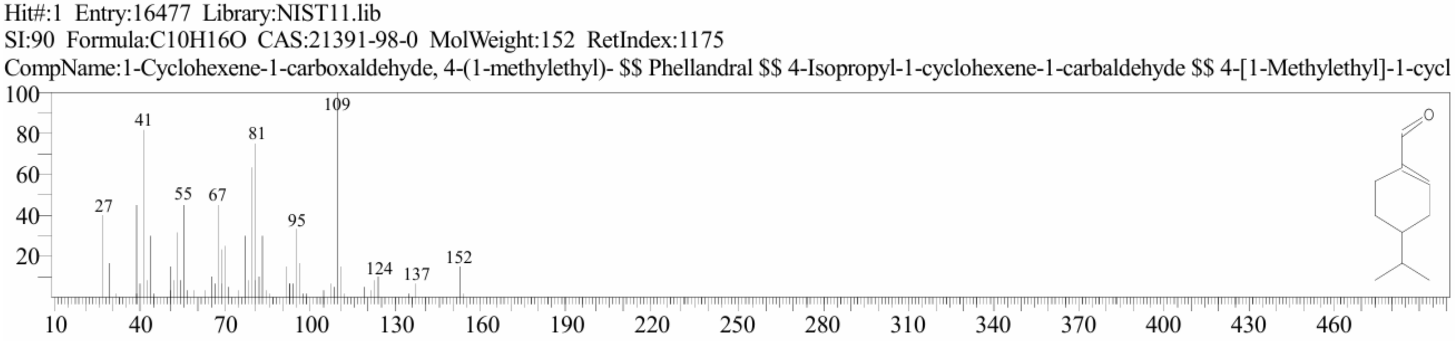

41	Bornyl acetate	Hit#:1 Entry:41489 Library:NIST11.lib<br>SI:97 Formula:C12H20O2 CAS:76-49-3 MolWeight:196 RetIndex:1277<br>CompName:Bornyl acetate $$ Bicyclo[2.2.1]heptan-2-ol, 1,7,7-trimethyl-, acetate, endo- $$ Borneol, acetate $$ Bornyl acetic ether $$ 2-Camphanol acetate<br>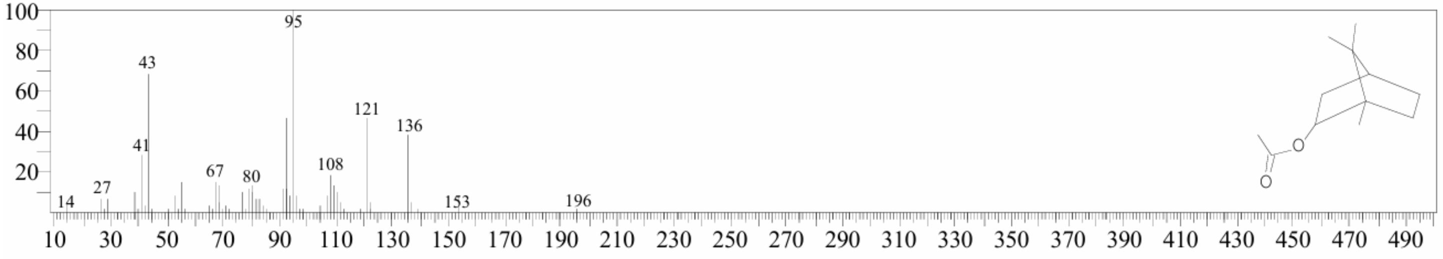
42	(+)-cis-Verbenol,<br><br>acetate	Hit#:1 Entry:20339 Library:NIST17s.lib<br>SI:90 Formula:C12H18O2 CAS:29135-27-1 MolWeight:194 RetIndex:1276<br>CompName:(+)-cis-Verbenol, acetate<br>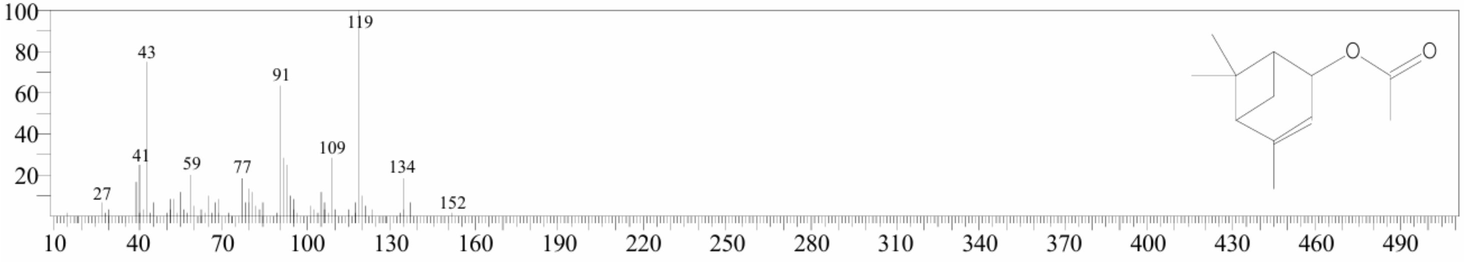

43	Shisool acetate	Hit#:1 Entry:46586 Library:NIST17-1.lib<br>SI:78 Formula:C12H20O2 CAS:89116-19-8 MolWeight:196 RetIndex:1374<br>CompName:Shisool acetate $$ (4-(Prop-1-en-2-yl)cyclohexyl)methyl acetate $$ Cyclohexanemethanol, 4-(1-methylethenyl)-, acetate $$ Cyclohexanemethar<br>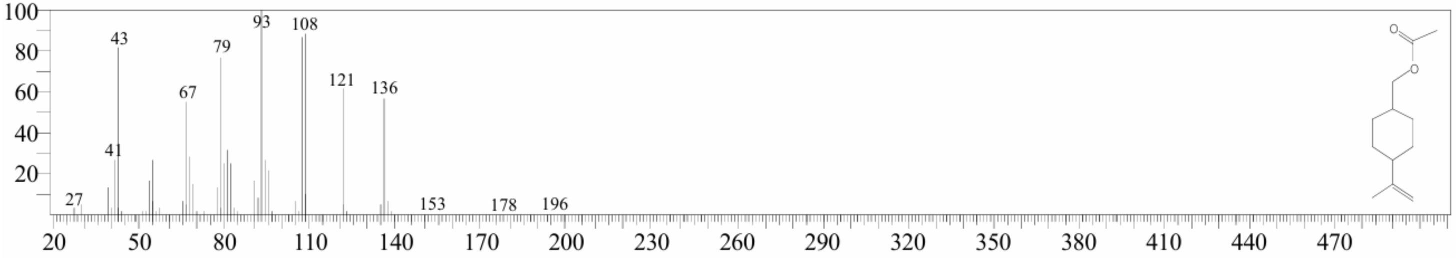
44	n-Hexadecanoic acid	Hit#:1 Entry:23313 Library:NIST11s.lib<br>SI:94 Formula:C16H32O2 CAS:57-10-3 MolWeight:256 RetIndex:1968<br>CompName:n-Hexadecanoic acid $$ Hexadecanoic acid $$ n-Hexadecoic acid $$ Palmitic acid $$ Pentadecanecarboxylic acid $$ 1-Pentadecanecarboxylic<br>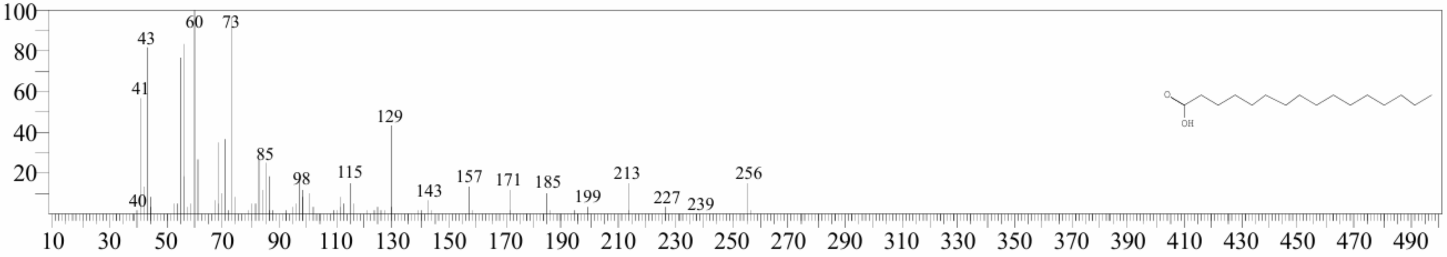

<table>
<tr><td>45</td><td>(R,1E,5E,9E)-1,5,9-Trimethyl-12-(prop-1-en-2-yl)cyclotetradeca-1,5,9-triene</td><td>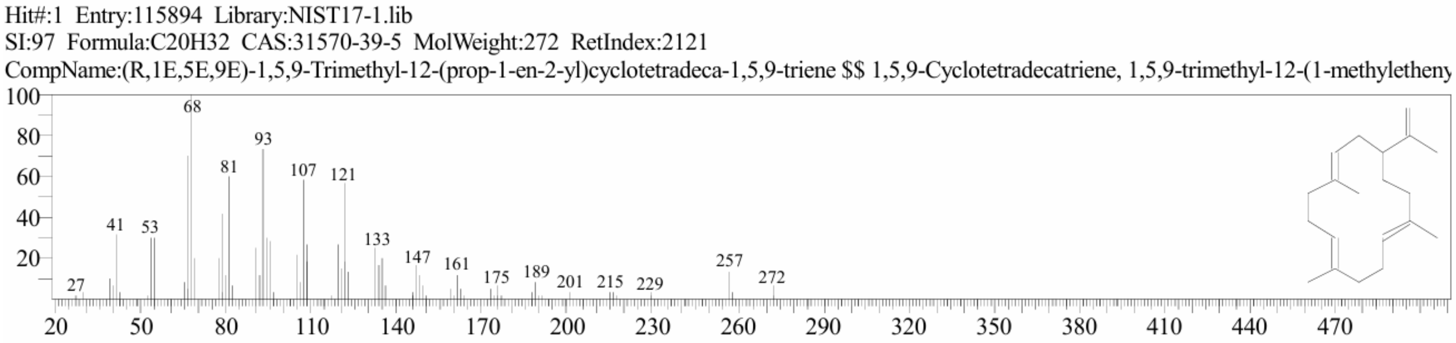
</td></tr>
<tr><td>46</td><td>Oleic Acid</td><td>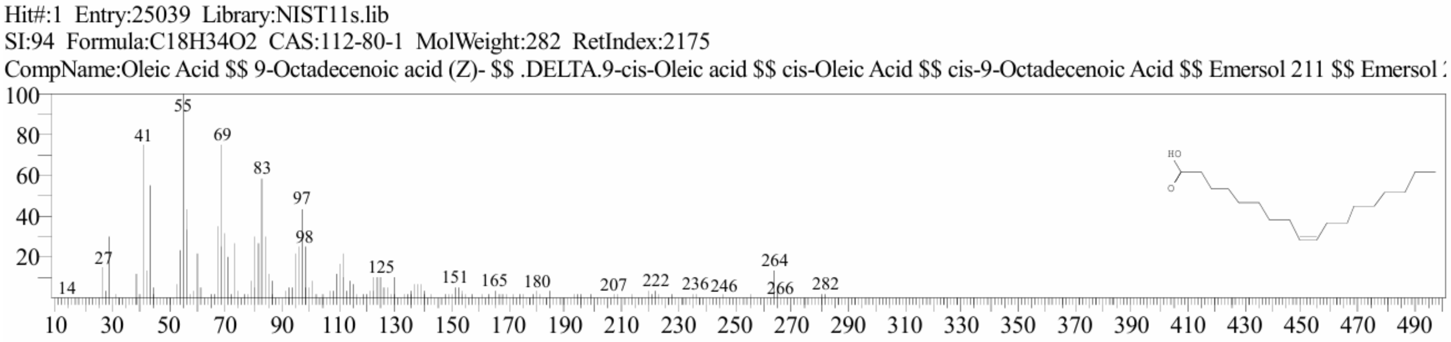
</td></tr>
</table>

47	Cycloheptane, 4-methylene-1-methyl-2-(2-methyl-1-propen-1-yl)-1-vinyl-	Hit#:5 Entry:46664 Library:NIST11.lib<br>SI:85 Formula:C15H24 CAS:0-00-0 MolWeight:204 RetIndex:1475<br>CompName:Cycloheptane, 4-methylene-1-methyl-2-(2-methyl-1-propen-1-yl)-1-vinyl- $$ 1-Methyl-4-methylene-2-(2-methyl-1-propenyl)-1-vinylcycloheptai<br>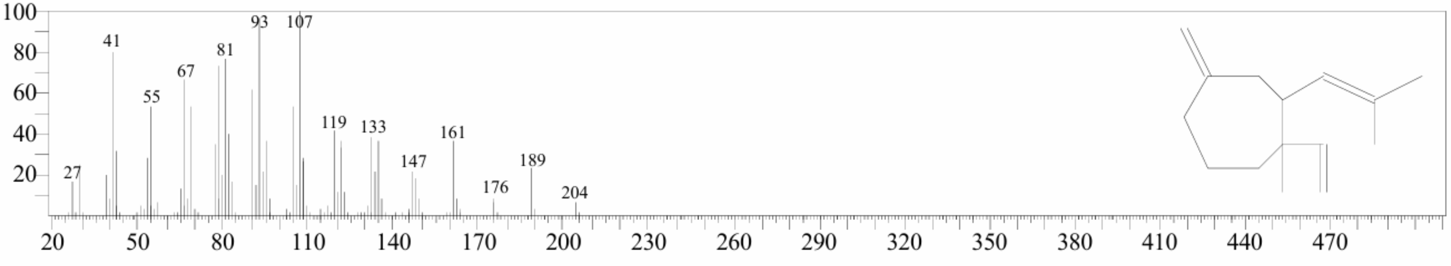
48	Incensole isomer	Hit#:1 Entry:150133 Library:NIST17-1.lib<br>SI:90 Formula:C20H34O2 CAS:0-00-0 MolWeight:306 RetIndex:2303<br>CompName:Isopropyl-1,5,9-trimethyl-15-oxabicyclo[10.2.1]pentadeca-5,9-dien-2-ol $$ Incensole isomer $$<br>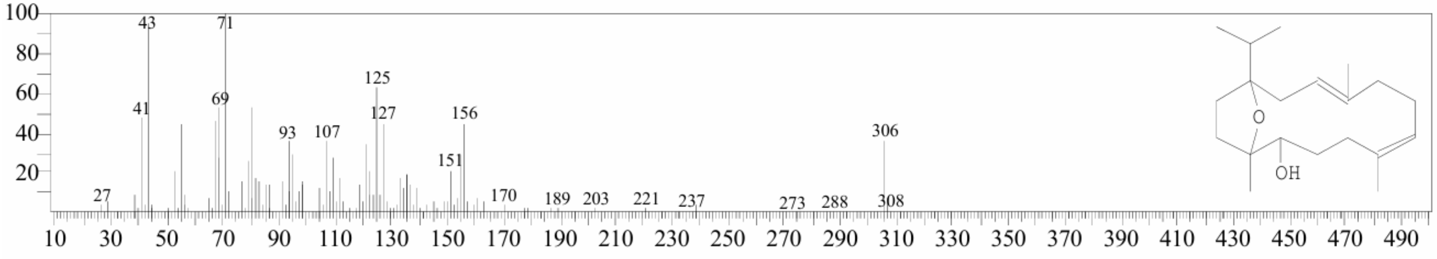
